# Screening ‘Gum Disease or Problem’ Status Using Molar Probing Depths: Model Development and Validation in NHANES Young to Middle-Aged Adults

**DOI:** 10.3390/dj14090560

**Published:** 2026-09-03

**Authors:** Miika Penttala

**Affiliations:** Department of Oral and Maxillofacial Diseases, Head and Neck Center, University of Helsinki and Helsinki University Hospital, 00290 Helsinki, Finland; miika.penttala@helsinki.fi

**Keywords:** automated clinical screening, decision curve analysis, electronic dental records, gum disease, molars, periodontitis, probing depth

## Abstract

**Background/Objectives:** A parsimonious multivariable model was engineered and externally validated using dental electronic health records to screen for a clinician-evaluated “gum disease or related problem” status. Designed for seamless electronic integration, a positive screening result triggers a targeted referral—and the model is well-suited for dental hygienist use— while providing clinical decision support for dentists during definitive diagnoses. This automated support standardises workflows, maximises team efficiency, and supports data-driven clinical decision-making. **Methods:** This cross-sectional study developed the model using the US National Health and Nutrition Examination Survey (NHANES) 2011–2012 cohort (*n* = 1732) and independently validated it on the 2013–2014 replication cycle (*n* = 1855) for the high-risk 30–50-year-old demographic. Survey-weighted logistic regression incorporated age, gender, and eight targeted molar probing depth (PD) measurements paired with binary indicators for tooth presence to predict whether an individual has a gum disease or related problem. **Results:** External validation demonstrated high parameter generalisability, yielding a pooled population-level AUC of 0.859 (95% CI: 0.828–0.885). At the prevalence-matched threshold of *Pt* = 0.367, the framework yielded a sensitivity of 78.39% (95% CI: 69.48–85.29%) and a specificity of 79.10% (95% CI: 74.55–83.04%). Demonstrating public health utility via Decision Curve Analysis, the model avoided approximately 39 unnecessary clinical examinations by a dentist per 100 individuals at a practical clinical decision threshold of 0.33, achieving a substantial pooled net benefit compared with a treat-all strategy without compromising case-detection metrics. **Conclusions:** This scalable screening model offers a highly practical pathway for gum disease and related problem interception. Optimised for seamless software integration, the framework facilitates rapid, hygienist-led risk stratification without increasing chairside burden, and delivers objective data streams to reinforce the dentist’s definitive diagnostic workflow.

## 1. Introduction

Many Nordic countries—such as Denmark, Finland, Norway, and Sweden—position dental hygienists as independent frontline screeners who conduct initial assessments and refer patients to dentists for definitive diagnosis. Structural efficiency and resource-saving divisions of labour sustain clinical capacity in these systems. Automated screening tools can further optimise these pathways, providing dentists with objective data that refines diagnostic decision-making. By operating beyond a rigid hygiene-to-dentist referral chain, such tools support an integrated, data-driven workflow that accelerates decisions and improves diagnostic consistency across the dental team [1,2].

The developed screening model predicts expert-validated “gum disease/problem” referral status by combining demographic data (age, gender) with eight targeted probing depth (PD) measurements on first and second molars. Within the studied 30–50-year-old NHANES derivation cohort, calibrated, licensed dentists established a baseline target condition prevalence of 36.7%, rendering a scalable triage tool paramount. This cohort-specific burden mirrors the wider epidemiological reality of this condition, as its advanced manifestation—periodontitis—ultimately affects nearly half of dentate adults aged 30 and older [3]. This widespread burden is a chronic, dysbiotic inflammatory disease triggered by dental plaque biofilms, leading to progressive destruction of the periodontium. Its clinical presentation characteristically includes gingival bleeding, pocket formation, progressive attachment loss, and radiographically detectable alveolar bone loss, eventually causing tooth mobility [4]. To address this efficiently, PD metrics offer greater speed, reproducibility, and lower technique sensitivity than Clinical Attachment Level (CAL), which requires locating the subgingival cementoenamel junction and inflates examiner variance [5]. Focusing on high-yield molar sites minimises charting burdens, supports early detection, and helps mitigate systemic inflammatory burdens [6]. The selection of molars for probing depth predictors was guided by their value as robust epidemiological indicators, owing to a complex anatomy and a high susceptibility to early tissue breakdown.

To ensure scalability without adding clinical burden, the tool leverages existing digital health records and parsimonious clinical findings. The model was developed using the NHANES 2011–2012 dataset and validated with the consecutive NHANES 2013–2014 cohort. Both datasets stem from a gold standard epidemiologic resource utilising a rigorous full mouth protocol, which calibrated, licensed dentists administered across a nationally representative U.S. population [7]. Questionnaire-based models using this dataset have predicted dental-visit need and guided self-assessment or primary-care triage [8]. In contrast, the present framework is optimised for clinical dental environments by incorporating direct, automated intraoral data streams.

Targeting the 30–50 age demographic for preventative screening is epidemiologically justified, as the incidence of periodontal disease increases significantly among young adults, peaking within the 45–49 age bracket [9]. Screening at this stage effectively prevents disease progression and severity. Methodologically, mean periodontal pocket depth remains stable across these decades [10]. Capturing this physiological window provides a precise baseline, ensuring maximum predictive accuracy before irreversible tissue destruction occurs.

Therefore, the aim of this study was to develop and externally validate a parsimonious, objective screening tool designed to predict expert-validated periodontal referral status in adults aged 30–50. From a scientific perspective, the objective was to evaluate the mathematical validity and clinical net benefit of a streamlined intraoral data stream, specifically testing whether a useful tool could be engineered using molar probing depths alone. From a clinical perspective, the aim was to establish a systematic triage filter for daily dental operations that minimises unnecessary evaluations for healthy individuals whilst ensuring robust, automated referral pathways for those requiring definitive diagnosis.

## 2. Materials and Methods

### 2.1. Study Design and Data Source

This cross-sectional investigation leveraged public-use files from the NHANES 2011–2014 waves. The primary development cohort was derived from the 2011–2012 cycle, whereas the consecutive 2013–2014 cycle provided an independent sample for temporal external validation [11]. The baseline data structure incorporated standardised full-mouth clinical periodontal examinations alongside self-reported questionnaires including core participant demographics, smoking behaviours, and historical dental utilisation patterns. Operating under the auspices of the NCHS Research Ethics Review Board, the original survey protocol obtained full ethical clearance, rendering this secondary analysis of de-identified records exempt from additional institutional oversight. All NHANES participants provided written informed consent prior to examination.

The licensed dentist-evaluated binary referral category for “gum disease/problem” classification served as the primary clinical outcome for screening utility. This metric represents a synthesised clinical judgment recorded by the examining licensed clinician (NHANES variable OHAROCGP, located in the OHXREF_G data file [12]). It divides participants into targeted categories requiring either definitive dentist referral or no further intervention based on objective oral health findings concerning gum disease or problems. This outcome specifically targets the broad spectrum of periodontal diseases, which are characterised as chronic or acute inflammatory conditions affecting the tooth-supporting tissues that can lead to irreversible bone and attachment loss.

In accordance with the NHANES Oral Health Examiners Procedures Manual, this referral recommendation was triggered by specific clinical thresholds, spanning routine, non-urgent care needs—such as moderate localised attachment loss or shallow pathological pocketing—to more advanced manifestations [13,14]. Reflecting established U.S. population-level epidemiological estimates for periodontal disease prevalence [15,16], this dentist-validated classification protocol yielded a referral prevalence of 36.7% within the primary derivation cohort (as detailed in Supplementary Materials: Figure S1, Table S1).

Operationally, the NHANES integrated software automatically processed the examiner’s clinical findings and measurements, generating the most severe referral code when multiple issues were recorded simultaneously. While preassigned software flags served as an automated baseline, the examining dentist retained full clinical override authority, making the final outcome assignment based on their expert professional judgment, accommodating both routine monitoring and urgent evaluations [13,14]. Participants who did not meet these distinct diagnostic criteria for immediate periodontal intervention were classified into the non-referral group. Comprehensive user interaction parameters, outcome variable diagnostics [17], and related descriptive characteristics are detailed in Supplementary Notes S1 and S2 and Table S2.

To ensure that these classifications were based on robust clinical foundations, data integrity was maintained through the rigorous quality control protocols built into the NHANES framework. To minimise measurement error, all dental examiners completed a mandatory, centralised 5-day training programme conducted by the NHANES reference examiner, which combined theoretical instruction with practical calibration sessions [13,14].

Standardisation initiatives yielded high consistency across examiners, allowing categorical periodontitis evaluations to achieve moderate-to-substantial inter-rater reliability with Kappa values ranging from 0.55 to 0.75 [7,13,14]. Similarly, continuous periodontal indicators—specifically clinical attachment levels and probing pocket depths—showed excellent absolute reproducibility, with inter-class correlation coefficients (ICCs) spanning from 0.79 to 0.90 [7]. All clinical evaluations strictly followed the operational criteria and standardised definitions detailed in the official NHANES Oral Health Examiners Manuals [13,14].

These rigorous metrics confirm that the underlying clinical data satisfy established quality thresholds for reliable population-level surveillance and predictive modelling. To ensure complete structural transparency and methodological rigor, the subsequent reporting and analytical phases were aligned with international guidelines. The overall observational research architecture adhered to the STROBE statement for cross-sectional studies [18], while the development, validation, and algorithmic workflows of the predictive framework strictly followed the TRIPOD + AI recommendations [19].

### 2.2. Participant Selection and Variable Specifications

#### 2.2.1. Participant Selection

**Figure 1:**
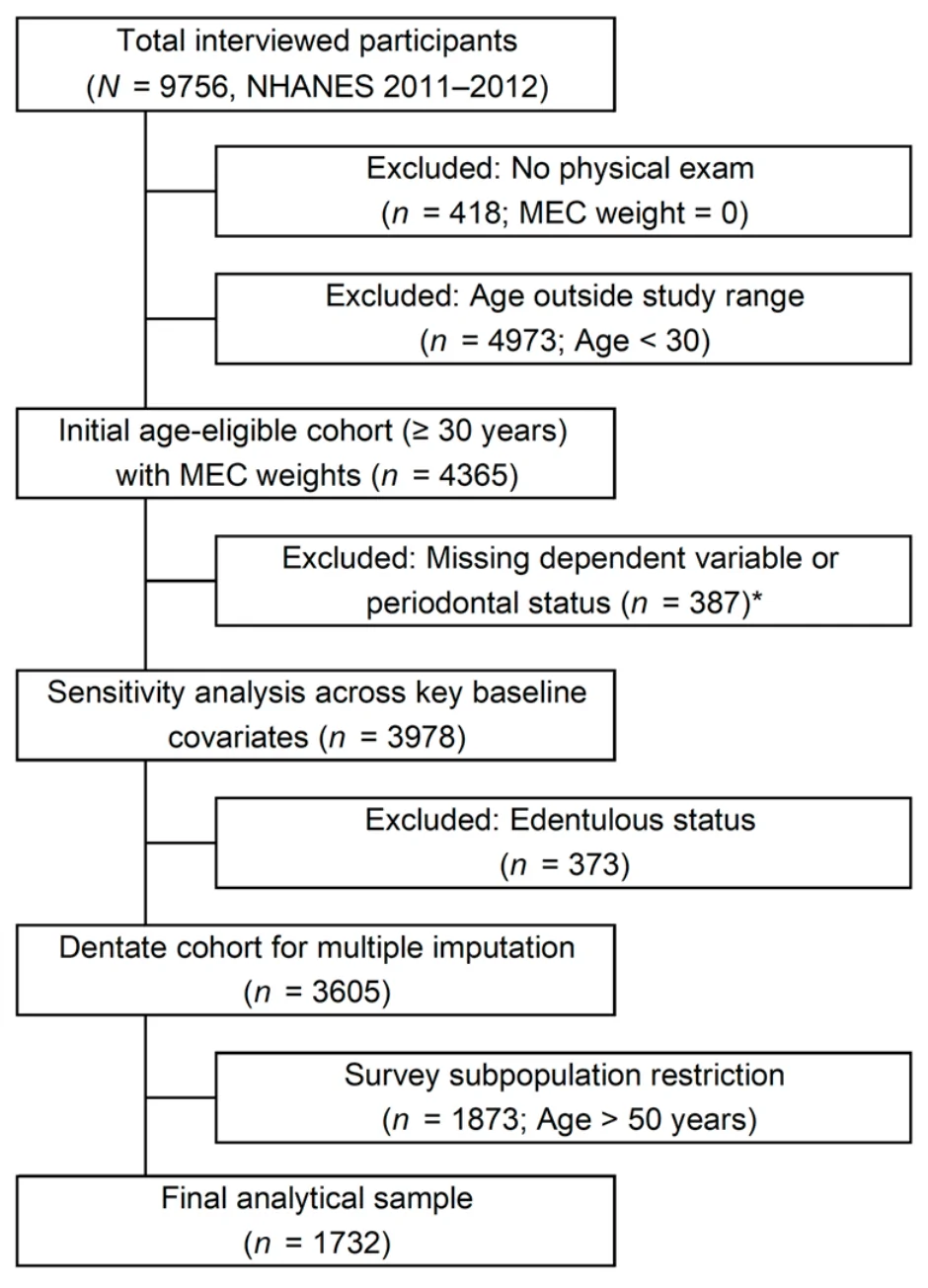
Participant selection and data restoration flowchart. The diagram shows the progression from initial NHANES 2011–2012 recruitment (*N* = 9756) through exclusion criteria, the baseline covariate sample (*n* = 3978), multiple imputation (*n* = 3605), and survey subpopulation restriction to account for complex survey design (*n* = 1873), resulting in the final analytic sample (*n* = 1732). * A comprehensive survey-adjusted non-response attrition analysis comparing the excluded subgroup (*n* = 387) against the remaining cohort (*n* = 3978) was executed to evaluate potential selection bias. Detailed stratification of missingness patterns, design-corrected statistical tests, effect sizes, and demographic parameters are fully detailed in Supplementary Materials: Note S3 and Table S3.

The analytic sample was derived from the initial NHANES 2011–2012 recruitment cohort (*N* = 9756). As illustrated in Figure 1, sequential exclusion criteria were applied: individuals who did not complete the physical examination component (*n* = 418) were first removed, followed by the exclusion of those under 30 years of age (*n* = 4973), thereby establishing a baseline age-eligible cohort (*n* = 4365). Within this cohort, 72 individuals missing data strictly for the dependent variable and 315 individuals lacking both the dependent variable and periodontal metrics (*n* = 387), were removed via listwise deletion to yield a baseline sensitivity sample (*n* = 3978). This conservative pre-imputation strategy follows Allison’s specific methodological framework, which recommends listwise deletion for cases with missing outcomes or complete absence of key predictors to prevent introducing severe imputation noise into the estimation equations [20].

Following the exclusion of edentulous individuals (*n* = 373), a dentate cohort of 3605 participants was assigned for multiple imputation. Finally, the survey subpopulation was restricted to target the younger age bracket by omitting participants aged over 50 years (*n* = 1873). This systematic selection cascade resulted in a final analytic sample consisting of *n* = 1732 dentate adults aged 30–50 years, providing a robust, nationally representative framework for reliable periodontal modelling (Figure 1; a detailed non-response attrition analysis accounting for NHANES non-response patterns [21] and analytical weight compensations [22] is provided in Supplementary Note S3 and Table S3).

#### 2.2.2. Probing Depth as the Primary Predictor

Probing depth (PD) was selected as the primary predictor for “gum disease/problem” status due to its efficiency, reproducibility, and suitability for large-scale epidemiological screening compared with clinical attachment level (CAL). As highlighted by Kingman and Albandar (2002), focusing on probing pocket depths rather than attachment loss is specifically indicated when the primary objective is to determine treatment needs and screen populations [23]. CAL charting requires localising the subgingival cementoenamel junction (CEJ)—a highly technique-sensitive procedure that compromises full-mouth reproducibility and becomes structurally unreliable under acute inflammatory conditions [3,21,22]. Because of these methodological constraints, and because examiner variance can inflate standard errors or bias model coefficients, PD was considered more practical than CAL for population-level assessments.

Furthermore, manual probing protocols used in epidemiological screening typically exacerbate variance due to 0.5 mm marking increments, while typical site-level measurement errors can be expected to fall between 0.38 and 0.50 mm [23,24]. This intrinsic unreliability stems from both manufacturing deficits—where tine markings deviate even within identical production batches—and operational inconsistencies in tip diameter, probing force, or angulation. The multi-step nature of CAL charting inevitably compounds these instrumental errors, generating substantial statistical noise [23,24]. Measurement utility, however, is also structurally maintained by local pathophysiology: pocket-base inflammation lowers tissue resistance to permit deeper probe penetration. Cascading analytical errors are inevitably introduced if CAL measurements are forced under equivalent inflammatory conditions, as such attempts lack the validation of increased tissue compliance that directly reflects active disease status [24].

Clinical equivalence between PD and CAL is further reinforced by the target population’s demographic profile. Among young and middle-aged adults (30–50 years), gingival recession is typically minimal or absent [10], meaning the gingival margin sits near the CEJ and PD accurately captures the full extent of periodontal destruction. Billings et al. (2018) confirm that while clinically meaningful recession increases with age, early-stage attachment loss remains concealed as a deepened pocket beneath a stable gingival margin in younger cohorts [10]. Consequently, targeting this age group is ideal because mean pocket depths remain stable across these decades, making PD a sufficient metric for early detection.

Taken together, these methodological and epidemiological considerations justify the use of PD as the forced predictor for “gum disease/problem” status in screening frameworks designed for streamlined, chairside implementation in routine dental practice.

Finally, within this study’s analytic framework, raw PD values were capped at 5 mm (yielding a discrete range of 0–5 mm). Because the model’s primary objective is screening, where clinical and diagnostic thresholds are typically met well below this limit, capping prevents extreme outliers from disproportionately influencing statistical coefficients while preserving the variance necessary for accurate prediction.

### 2.3. Statistical Analysis

#### 2.3.1. Data Imputation and Survey Design

All statistical analyses were conducted using R software (version 4.6.1; R Foundation for Statistical Computing, Vienna, Austria). The predictive model developed for this research utilised age, gender, and eight probing depth (PD) measurements—alongside molar presence—as clinical inputs. These eight measurements were selected from an initial pool of 32 candidate clinical predictors across all eight permanent first and second molars (one site per tooth). Missing data, mainly concerning this initial pool of 32 candidate PD sites, were addressed using fully conditional specification (FCS) via the mice package (version 3.19.0) [25,26], generating 20 complete datasets. To enhance the quality of the imputation, the FCS models also incorporated 10 auxiliary variables—such as smoking, diabetes status, and oral health pattern variables—which exhibited minimal missingness but provided essential informational context without participating in the subsequent model selection. A detailed, step-by-step description of the survey-adjusted imputation protocol is provided in Supplementary Note S4, and the complete missing data patterns and variable profiles are summarised in Supplementary Tables S4 and S5.

Data leakage was avoided by processing the development (NHANES 2011–2012; *n* = 1732) and independent validation (NHANES 2013–2014; *n* = 1855) cohorts separately [13]. The imputation model explicitly accounted for the complex NHANES design by deploying a single integrated cluster variable—constructed from the interaction of survey strata (SDMVSTRA) and primary sampling units (SDMVPSU)—alongside the Mobile Examination Center sample weights (WTMEC2YR) directly within the conditional drawing loops [27].

It is important to note that the PD measurements within the analytic framework of this modelling study ranged from 0 to 5 mm. In alignment with the overall study protocol, raw PD measurements exceeding 5 mm were capped at 5 mm to maintain statistical stability, thereby restricting the PD measurements strictly to values of 0, 1, 2, 3, 4, or 5 mm (the operational coding and imputation mechanics are detailed in Supplementary Note S4).

#### 2.3.2. Predictor Selection and Model Development

**Figure 2:**
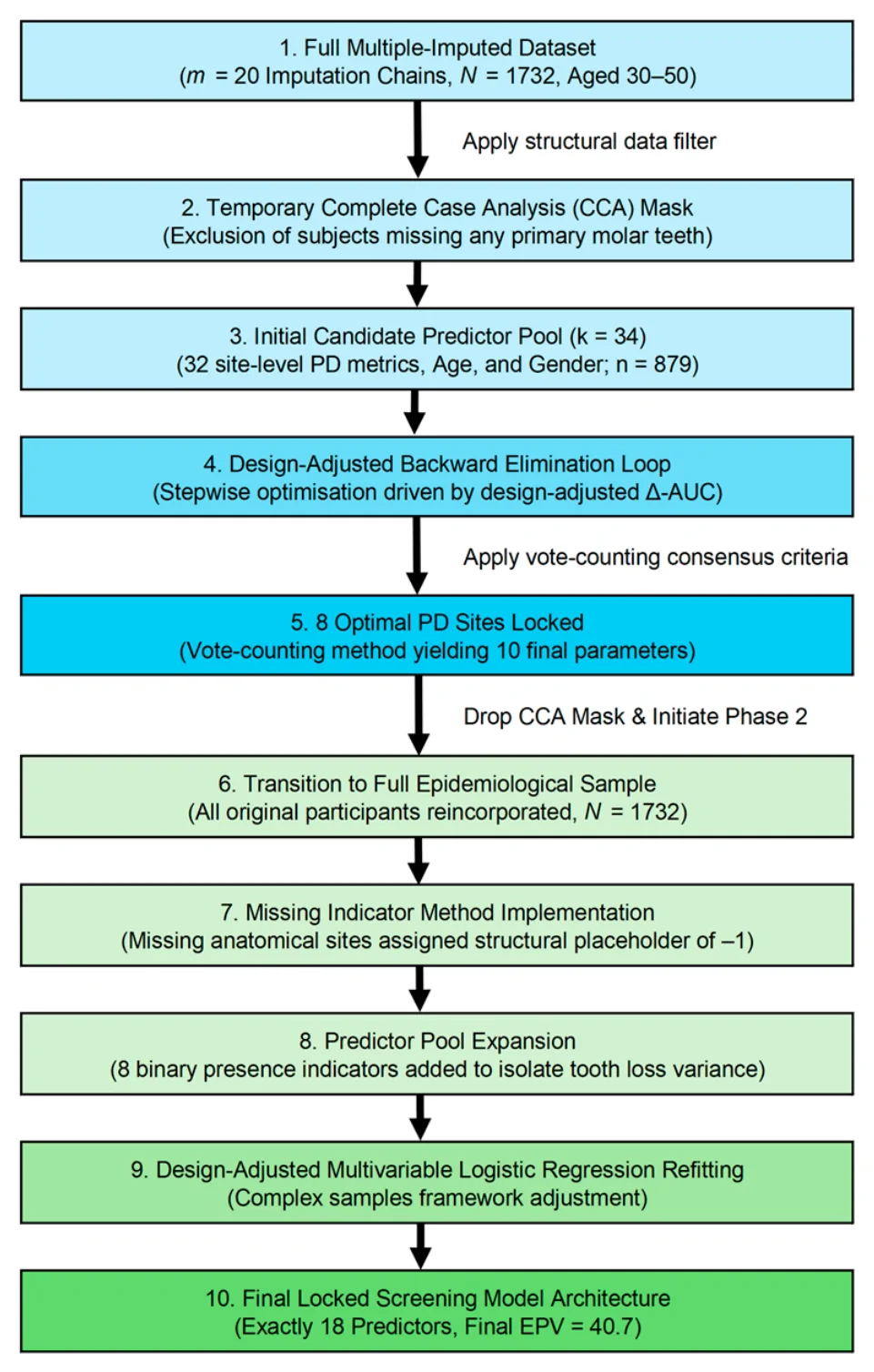
Two-phase algorithmic framework for predictor selection and model development. Phase 1 (Boxes 1–5) illustrates the localised Complete Case Analysis (CCA) framework implemented to isolate a clean biological probing depth (PD) signal, utilising backward elimination across the CCA sub-sample (*n* = 879) to select 8 optimal anatomical sites (yielding 10 initial parameters including age and gender). Phase 2 (Boxes 6–10) depicts the transition back to the full epidemiologically weighted dataset (*N* = 1732), incorporating structurally missing positions via the Missing Indicator Method and expanding the pool with 8 binary indicators to isolate tooth loss variance, resulting in the final locked 18-predictor screening tool.

The screening model was engineered using a structured, two-phase feature selection framework to isolate high-yield clinical indicators before final multivariable regression modelling (Figure 2) [28].

Phase 1 deployed a temporary Complete Case Analysis (CCA) framework restricted solely to the exploratory variable selection loop. To isolate a clean biological probing depth (PD) signal and prevent mathematical artifacts driven by tooth loss, participants with any missing primary molars within the candidate sites were temporarily excluded via listwise deletion. This localised restriction yielded a fully dentate training subset (*n* = 879; unweighted outcome prevalence: 30.0%) to evaluate an initial pool of 34 candidate predictors (*k* = 34: comprising 32 site-level continuous PD metrics from the eight permanent molars, age, and gender).

A global backward elimination procedure was executed independently within each of the 20 imputed datasets using design-adjusted logistic regression models via the “survey” package. Continuous PD variables were systematically removed based on the smallest step-by-step reductions in the design-adjusted Area Under the ROC Curve (AUC), until exactly eight optimal PD variables remained alongside age and gender. Final parameters were locked based on a vote-counting method across the 20 imputed datasets, keeping only the variables that were selected most frequently (algorithmic parameters and tie-breaking criteria are detailed in Supplementary Note S5).

In Phase 2, the architecture transitioned back to the full epidemiologically representative sample (*n* = 1732). To structurally account for missing teeth without dropping data, the Missing Indicator Method was deployed. Missing anatomical sites were assigned a continuous placeholder value of −1 and paired with an explicit binary presence/absence indicator variable (0 = missing, 1 = present). This dual-variable configuration isolated the structural variance of tooth loss, preventing coefficient distortion or attenuation bias. The finalised screening model comprised 18 predictors: eight optimal continuous PD sites, eight binary tooth presence indicators, age, and gender (details are provided in Supplementary Table S6).

#### 2.3.3. External Validation and Performance Evaluation

Due to the availability of two large, distinct national waves of the NHANES database (*n* = 1732 for the 2011–2012 training cohort and *n* = 1855 for the 2013–2014 validation cohort), internal cross-validation was bypassed in favor of a more rigorous external validation. Because all 18 predictors (8 continuous molar PD measurements, 8 binary molar PD measurements, age, and gender) were clinically pre-specified, internal data-splitting or cross-validation for variable selection was unnecessary. Direct external validation avoided the loss of sample size and statistical power within the complex survey design of the training cohort, while providing a robust, unbiased evaluation of the model’s transportability to an independent population. Model transportability was subsequently assessed by applying the fixed baseline parameters directly to the independent validation cohort. Performance evaluations across all statistical dimensions fully accounted for the multi-stage clustered survey design and multiple imputation variance. Discrimination was evaluated using the design-adjusted C-statistic, quantified via the Area Under the Curve (AUC), while calibration was comprehensively quantified at both group-level deciles and individual-level parameters using the design-adjusted calibration slope, de-sign-adjusted intercept, and design-adjusted Brier score.

Clinical utility was quantified via Decision Curve Analysis (DCA) tailored for complex survey data by calculating population-weighted true-positive and false-positive fractions to estimate the pooled Net Benefit across a practical threshold range [29]. Final classification metrics, including design-adjusted sensitivity and specificity, were evaluated at the selected risk threshold to anchor this specific decision context, with all pooled estimates derived using Rubin’s rules. This framework ensures that all reported standard errors fully reflect the joint variance introduced by both the complex survey architecture and the multiple imputation process. The model implementation workflows, full model specification, model updating details, cut-off specifications, and the clinical and mathematical justifications for the selected decision threshold are provided chronologically in Supplementary Materials: Note S6, Note S7, Note S8, Note S9, and Note S10, respectively.

## 3. Results

### 3.1. Sample Characteristics

Age and gender were included as fixed a priori predictors, and eight tooth-specific PD variables were selected from 32 candidate interproximal surfaces (four per molar for eight molars). Sequential feature elimination and backtracking produced a final eight-site anatomical architecture: C02A, S03D, S14D, C15A, C18A, S19S, C30P, and C31P. Five sites were lingual (C) and three buccal (S), with directional orientation evenly split between distal (D, P) and mesial (S, A). These sites were incorporated alongside binary molar presence indicators directly into the multivariable prediction model (Table 1). Pooled mean PD values across these targeted loci were consistently higher among individuals with gum “disease/problem” status, forming the baseline contrast for subsequent modelling.

The population-weighted prevalence of gum disease or related problem in the training cohort was 36.7%. Using NHANES weights, the analytic sample (*n* = 1732) remained representative of dentate U.S. adults aged 30–50 years (Table 1). Tooth retention analysis showed Tooth 19 had the lowest presence rate (79.7%), dropping to 67.9% in the diseased subgroup, closely followed by Tooth 30 at 69.5%.

Demographic contrasts were also pronounced. Males comprised 62.7% of the diseased subgroup despite representing 49.9% of the full sample, while females were more prevalent in the healthy cohort (57.5%). Mean age increased steadily across groups: diseased individuals averaged 41.0 years (SD = 5.9) compared with 39.6 years (SD = 6.3) in the disease-free subgroup.

Expanded population-weighted distributions of sociodemographic, chronic health, and dental characteristics are provided in Supplementary Table S7.

### 3.2. Multivariable Model and Predictor Performance

The multivariable logistic regression model of the NHANES data strongly predicted the licensed dentist-diagnosed gum disease or related problem. When deployed within an external validation cohort (*n* = 1855), the 18-variable model (comprising 8 tooth-specific joint pairs and 2 demographic covariates) demonstrated robust discriminative capacity, achieving a pooled C-statistic (AUC) of 0.859 (95% CI: 0.828–0.885). Operating at the predefined clinical threshold cutoff of 0.367—strategically selected to match the training cohort prevalence and prioritise population-level case detection (Supplementary Materials: Figure S1, Note S9)—the screening framework yielded a highly balanced diagnostic trade-off, characterised by a sensitivity of 78.39% (95% CI: 69.48–85.29%) and a specificity of 79.10% (95% CI: 74.55–83.04%).

**Figure 3:**
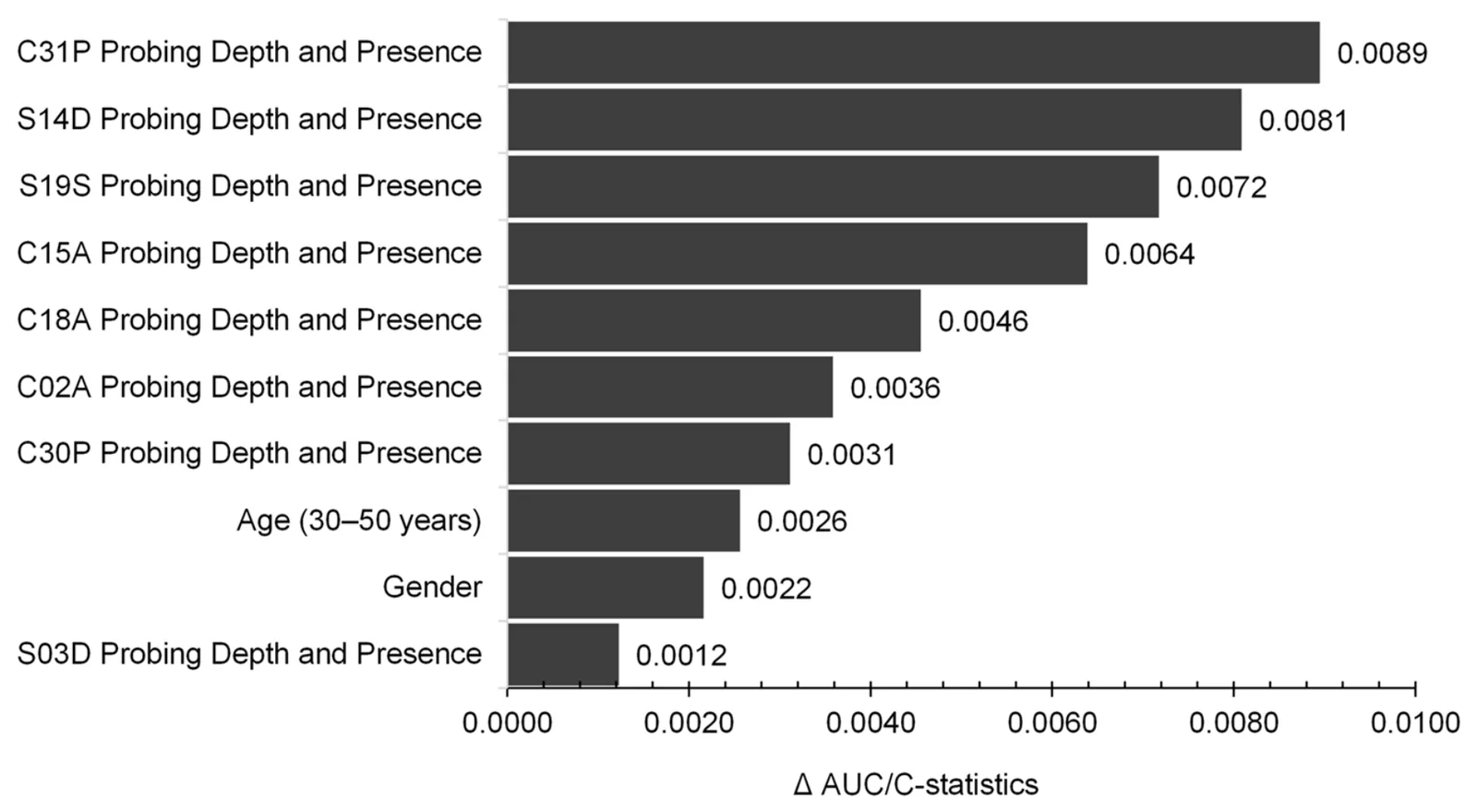
Relative feature contributions to model predictive power. Bars represent the unique contribution of each predictor or tooth-specific block to the total Area Under the Curve, quantified as the incremental decrease in AUC (ΔAUC) upon feature removal from the full multivariable framework. Model features include demographic covariates (age and gender) and joint tooth-specific pairs comprising binary tooth presence and its corresponding clinical probing depth (representing 16 tooth-specific variables and 2 demographic covariates, totalling the 18 predictors detailed in Figure 2). Tooth-specific alphanumeric codes correspond to the dental notation detailed in Table 1. ΔAUC estimates are averaged across the 20 multiple-imputed datasets, relative to a full training model baseline AUC of 0.894. External validation performance is detailed in Supplementary Table S8.

To evaluate which variables contributed the most to this predictive accuracy, a feature importance analysis was conducted using model reduction (Figure 3). When features were systematically extracted from the full framework, the exclusion of the C31P block triggered the sharpest independent drop in predictive power (ΔAUC = 0.0089), followed closely by the S14D (ΔAUC = 0.0081) and S19S (ΔAUC = 0.0072) joint pairs. Interestingly, the broader covariates of age (ΔAUC = 0.0026) and gender (ΔAUC = 0.0022) cluster toward the bottom of the horizontal hierarchy, outperforming only the S03D joint pair (ΔAUC = 0.0012). This structural positioning confirms that while demographic factors remain statistically adjusted for model calibration, the diagnostic weight and operational sensitivity of the screening architecture are primarily driven by specific pocketing and tooth presence dynamics.

When Figure 3 results are compared with the crude regression results using the Nagelkerke R^2^ values, a broadly consistent trend is observed. An examination of the crude bivariable regression results (Supplementary Table S9) reveals that the joint tooth-specific pair for Tooth 15 was identified as the strongest predictor with a Nagelkerke R^2^ of 0.284, followed by the pairs for Tooth 31 (R^2^ = 0.280), Tooth 14 (R^2^ = 0.274), Tooth 02 (R^2^ = 0.274), and Tooth 19 (R^2^ = 0.268), whereas the weakest predictive blocks were Tooth 03 (R^2^ = 0.232) and Tooth 30 (R^2^ = 0.218). It is noteworthy that in these pairwise bivariable models, tooth retention and active tissue loss operate as an interconnected clinical balance. While tooth presence provides an initially low baseline risk, a 6-step incremental shift into a high-severity diseased state completely overrides this protection—elevating the overall screening risk 6-fold compared to an absolute extraction reference (as detailed in the Supplementary Table S9 interpretation text).

The adjusted model achieved a robust pooled Nagelkerke pseudo-R^2^ of 0.593 across the 20 multiple-imputation cycles within the training set (Table 2).

Furthermore, a comparison between the fully adjusted and the univariable (crude) models revealed stable behavioural profiles for the demographic predictors. In the univariable analysis (Supplementary Table S9), male gender exhibited a strong, unadjusted association with the outcome (OR = 2.269, 95% CI: 1.769–2.911, *p* < 0.001), which remained highly significant and stable after multivariable adjustment (aOR = 2.098, 95% CI: 1.495–2.945, *p* < 0.001, Table 2). Similarly, age proved to be a robustly independent baseline risk factor; its continuous effect size shifted only minimally from the crude model (OR = 1.038, 95% CI: 1.012–1.064, *p* = 0.004) to the final multivariable framework (aOR = 1.034, 95% CI: 1.006–1.062, *p* = 0.015). Because age is modelled as a continuous function, its independent risk scales exponentially over time. Within the targeted 30–50 years demographic, this 20-year age gap yields a cumulative odds ratio of 1.952, making a lifespan perspective clinically comparable to the fixed, nearly two-fold risk associated with male gender. When these two independent risk factors are combined, the joint mathematical effect escalates. Consequently, a 50-year-old male exhibits approximately four-fold higher adjusted odds of having a “gum disease/problem” status compared to a 30-year-old female.

Analytically, determining the primary or most influential clinical predictor depends on the operational criteria applied to the tooth-specific blocks due to the conditional adjustment between tooth presence and probing depth. Table 2 illustrates that if the primary criterion is the magnitude of risk escalation driven by pocketing severity, the C15A continuous metric emerges as the most potent clinical indicator, doubling the adjusted odds of the outcome (aOR = 2.056, 95% CI: 1.369–3.087, *p* < 0.001) among present teeth (T15). Alternatively, if predictive prominence is defined by the total dynamic range—capturing both a near-maximum protective baseline and a steep risk trajectory—the Tooth 19 joint block serves as the most balanced and comprehensive clinical anchor in the model (T19: aOR = 0.093, 95% CI: 0.032–0.270, *p* < 0.001; S19S: aOR = 1.821, 95% CI: 1.315–2.521, *p* < 0.001). Direct ranking between the Tooth 19 and Tooth 31 joint blocks presents a statistical trade-off; the T31 binary indicator offers a marginally more potent protective constraint (unrounded aOR = 0.0928 vs. 0.0934) than T19, whereas the S19S continuous parameter exhibits a sharper risk escalation upon pocketing than C31P (aOR = 1.821 vs. 1.739, *p* < 0.001).

### 3.3. Model Calibration

**Figure 4:**
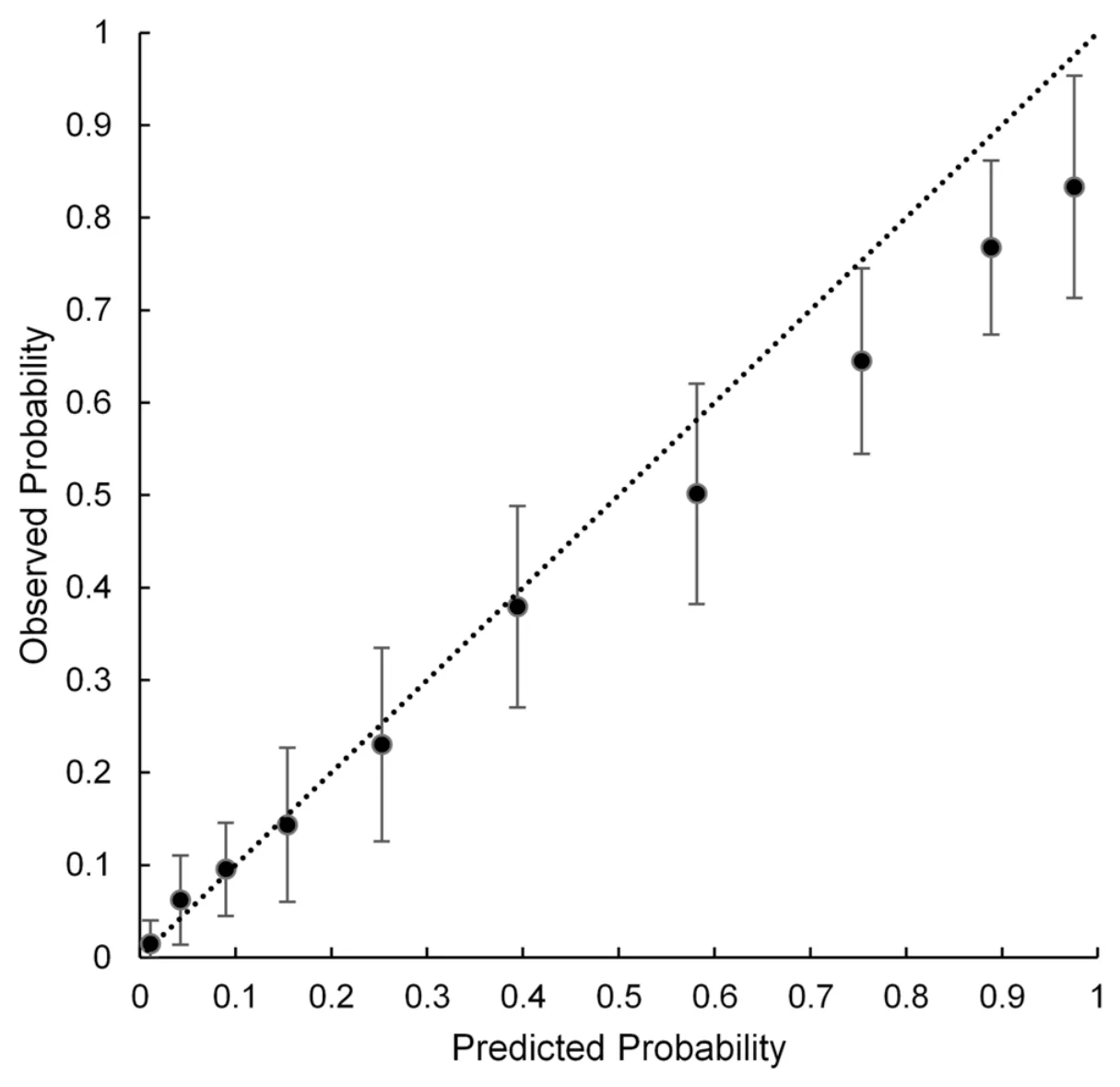
Risk calibration and decile-specific agreement within the external validation cohort (NHANES 2013–2014). Points and vertical error bars indicate pooled observed versus predicted probabilities across risk deciles with 95% confidence intervals, adjusted for complex survey design and multiple imputation variance. The dashed diagonal line represents ideal calibration (y = x). The model demonstrated stable calibration performance across the population spectrum, yielding a pooled calibration slope of 0.699 (95% CI: 0.536–0.862), and an intercept of −0.407 (95% CI: −0.672 to −0.142).

External calibration metrics reinforced the geographic and temporal stability of the predictive architecture across the full complex survey spectrum (Figure 4). The pooled calibration slope was established at 0.699 (95% CI: 0.536–0.862), reflecting a characteristic calibration attenuation typical of independent temporal validation. A minor baseline over-prediction of risk was observed when averaged across the entire survey population, as reflected by a slightly negative calibration intercept of −0.407 (95% CI: −0.672 to −0.142). The overall predictive accuracy and accuracy of probabilistic forecasts were confirmed by a design-adjusted, pooled complex samples Brier score of 0.137 (95% CI: 0.122–0.153), verifying that the locked framework retains high calibration precision and minimal forecast error when translated to an independent validation wave.

Decile-based risk mapping confirmed tight convergence between empirical observations and algorithmic predictions, with the ideal 45-degree reference line running directly through the 95% confidence envelopes of the majority of target strata. Excellent algorithmic alignment was sustained across low-to-moderate risk levels up to a predicted probability threshold of approximately 0.39 (Deciles 1–6). Beyond this risk boundary, a systematic divergence from the diagonal reference line emerged due to model over-confidence in the higher disease spectrum; specifically, within the tenth risk decile, a predicted probability of 97.55% corresponded to an observed clinical periodontitis prevalence of 83.29%.

### 3.4. Model Diagnostics and Assumptions

Model diagnostics indicated that the multivariable framework was stable and rigorously met all underlying statistical assumptions. Multicollinearity was assessed across the imputed datasets using Variance Inflation Factors (VIF), yielding a maximum observed VIF of 7.67. While this index reflects the expected structural correlation between continuous probing depth metrics and their corresponding binary tooth-presence indicators when retaining the conditional value of −1, the variance inflation remained safely below the standard critical threshold of 10.0, thereby confirming that multicollinearity did not compromise the validity of the regression estimates. 

Case-wise diagnostic screening pooled across all 20 imputed datasets according to Rubin’s rules revealed that only a minor proportion of observations exhibited large residuals. Averaged across all imputations, 2.06% of observations presented standardised residuals greater than 3.0, and 0% presented deviance residuals greater than 3.0. Crucially, the absolute maximum Cook’s Distance across all imputations was exceptionally low (0.085), remaining well below the conventional threshold of 1.0, which indicates that no single observation or imputation-specific artifact exerted undue influence on the pooled model estimates.

Furthermore, model development achieved a robust raw events-per-variable (EPV) ratio of 41 (based on 732 unweighted events and 18 model parameters), substantially exceeding the recommended minimum threshold of 10 [30]. The structural stability and transparent representation of the continuous and binary indicators across the imputed datasets ensured full regression model validity, which was further supported by a Box-Tidwell test confirming that the linearity assumption was met for all continuous variables (*p* > 0.05).

### 3.5. Sensitivity Analysis and Model Robustness

To evaluate the structural stability and reproducibility of the 18-variable screening architecture, comprehensive sensitivity analyses were performed across data-handling methodologies and independent population cohorts.

Descriptive analysis confirmed that the baseline population-weighted gum disease/problem prevalence was highly stable across temporal windows, yielding 36.71% (95% CI: 31.02–42.82%) in the development cohort (2011–2012) versus 32.69% (design-adjusted 95% CI: 27.69–38.16%) in the independent validation cohort (2013–2014), with the substantial confidence interval overlap demonstrating that the prevalence rates are statistically indistinguishable (Supplementary Figure S1). This equilibrium provided an unconfounded foundation for robust external validation.

Model resilience was first challenged using an unconstrained, fully re-estimated multivariable framework within the independent NHANES 2013–2014 replication cohort (*n* = 1855). The unconstrained model achieved a robust pooled Nagelkerke pseudo-R2 of 0.501 across 20 multiple-imputation cycles, exhibiting expected, minimal shrinkage from the 0.593 recorded during development. Perfect directional consistency of all 18 predictors was preserved under temporal translation, confirming sufficient mathematical stability.

Furthermore, benchmarking primary multivariable estimates against a complete-case analysis subset (*n* = 1546) demonstrated 100% directional consistency and tight parameter alignment, confirming that the primary imputation framework introduced no systemic mathematical distortions (Supplementary Table S10). While minor shifts in statistical significance levels occurred for specific loci such as Tooth 30 due to the integration of missing observations, the numerical trajectories of the adjusted odds ratios (aOR) remained highly secure. A detailed, granular breakdown of these cohort-specific parameter drifts, complete-case numerical translations, and individual feature performance fluctuations is provided in the Supplementary Note S11.

### 3.6. Clinical Utility and Net Benefit

**Figure 5:**
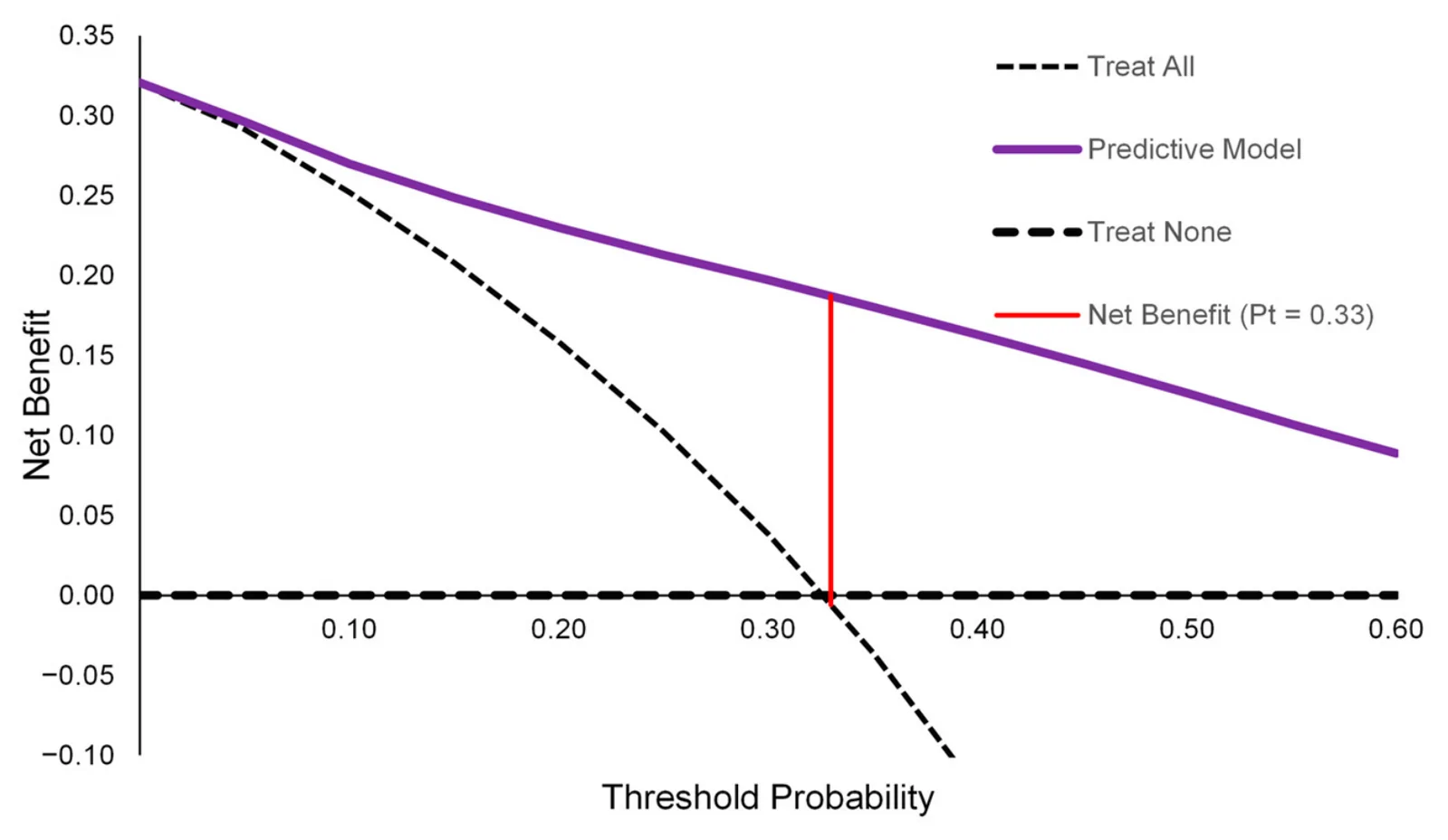
Decision curve analysis in the external validation dataset (NHANES 2013–2014, *n* = 1855). The hybrid model outperformed the default treat-all and treat-none strategies across all clinically relevant risk thresholds. At the selected decision threshold probability (*Pt*) of 0.33, the model yielded a net benefit of 0.187 compared to −0.005 for the treat-all strategy, achieving an absolute net-benefit gain of 0.192. This clinical optimisation successfully prevents approximately 39 unnecessary population screenings per 100 individuals without sacrificing overall case detection sensitivity.

External validation using the independent NHANES 2013–2014 cohort (*n* = 1855) demonstrated that the model provides a higher net benefit compared to both the default treat-all and treat-none strategies, as shown in the decision curve analysis in Figure 5. The predictive framework maintains this distinct clinical utility across the entire range of evaluated threshold probabilities (*Pt* ≥ 0.00), confirming its robust operational value when applied to an independent population wave.

At the targeted decision threshold of *Pt* = 0.33—selected to optimise the balance between diagnostic yield and resource allocation—the model achieved a pooled net benefit of 0.187 compared with a negative net benefit of −0.005 for the default treat-all strategy. This absolute improvement (ΔNB = 0.192) corresponds to avoiding approximately 39 unnecessary clinical screenings per 100 individuals without compromising case detection, mathematically calculated via the standard net benefit decision-analytic framework.

The model’s diagnostic advantage persisted and expanded at higher clinical risk thresholds. For example, at *Pt* = 0.40, the net benefit for the hybrid framework remained highly positive at 0.163, whereas the default treat-all strategy plummeted to −0.122, and the treat-none strategy remained static at zero.

Further examination of the external cohort demonstrated that the *Pt* = 0.33 threshold provided an incremental improvement in diagnostic sensitivity compared with the alternative baseline *Pt* = 0.367 prevalence cutoff. Specifically, screening sensitivity for gum disease and related problems increased to 80.50% (95% CI: 72.08–86.89%), up from the 78.39% (95% CI: 69.48–85.29%) recorded at the 0.367 level. Full breakdowns of these threshold trade-offs are provided in Supplementary Note S10.

## 4. Discussion

Oral examinations conducted by calibrated, licensed dentists within the NHANES framework ensure high clinical validity and population-level generalisability. Building upon this dataset, this study developed a parsimonious screening tool to predict the dentist-evaluated gum disease or related problem status. The streamlined 8-site probing depth (PD) model architecture achieved a robust pooled external validation AUC of 0.859 (95% CI: 0.828–0.885).

To establish clinical placement, this framework must be evaluated against existing prediction paradigms and contemporary clinical decision-support systems (CDSS) regarding structural complexity, operational environments, and clinical utility. Unlike classic maintenance frameworks such as the Periodontal Risk Assessment (PRA) tool [31], which demands full-mouth periodontal charting and radiographic logs, this model utilises only eight targeted molar probing depths and their binary tooth-presence indicators alongside baseline age and gender covariates. This mathematical parsimony shifts clinical utility from a time-consuming examination to a rapid screening workflow. Furthermore, while public health initiatives historically turned to subjective, interview-based self-reporting [32] to bypass resource-intensive clinical examinations—a method fundamentally limited by low sensitivity for early-stage disease—this framework relies on objective, automated clinical metrics restricted to optimal molar sites to balance diagnostic accuracy with minimal chairside burden.

Beyond classic dental frameworks, this clinical workflow offers distinct advantages over contemporary medical machine learning models and radiographic software. For instance, an XGBoost classifier [33] incorporates 77 systemic input features spanning haematologic markers and heavy metal toxicology to catch periodontitis in primary care environments. While innovative as a non-dental risk calculator, that model remains impractical for routine dental practice deployment due to its limited performance (precision 57.4%, accuracy 61.1%), unmeasured behavioural confounding from hidden lifestyle metrics, and the necessity of invasive blood laboratory testing.

Currently, dental decision-support platforms are dominated by commercial computer vision and machine learning algorithms designed to automatically detect dental caries, periapical pathologies, and marginal bone loss directly from intraoral and panoramic radiographs [34,35]. While these visual AI tools provide valuable diagnostic secondary opinions for dentists, they operate strictly as localised image-interpreters rather than integrated clinical workflow orchestrators. Conversely, non-radiographic electronic decision-support systems, such as automated Caries Risk Assessment (CRA) modules based on digital CAMBRA frameworks [36] or the computer-based Periodontal Risk Calculator (PRC) [37]—often face implementation barriers due to the heavy reliance on time-consuming, manual data entry by clinicians. In biomedical informatics, this reliance on continuous manual input within poorly integrated user interfaces drives clinical ‘documentation burden’ and cognitive fatigue, leading to the underutilisation of risk calculators in high-volume public dental health clinics [38].

The screening model presented here addresses this specific operational gap. Rather than acting as a static risk calculator, it functions as an automated triage mechanism embedded directly within standard electronic dental and health records (EDR/EHR). By processing data seamlessly in the background as the clinician works, the tool substantially reduces data-entry friction. Crucially, whereas prior epidemiological and machine learning models are evaluated solely on statistical accuracy metrics, real-world clinical and economic utility is validated here via Decision Curve Analysis (DCA). At a practical clinical threshold (*Pt* = 0.33), this parsimonious model demonstrates the potential to safely avoid approximately 39 unnecessary comprehensive dentist examinations per 100 individuals without compromising case-detection metrics (Figure 5). While this projected 39% reduction represents a theoretical optimisation derived from retrospective cohorts, it establishes a compelling baseline for future prospective trials to measure actual structural workload shifts.

The clinical efficacy of this screening utility stems directly from its underlying anatomical design. When individual molar contributions were deconstructed during anatomical analysis (Figure 3), the top risk drivers exhibited a distinct geometric counterbalance between the mandibular and maxillary arches. This architectural equilibrium is structurally achieved by balancing three buccal and five lingual molar parameters alongside an equal distribution of four mesial and four distal sites. By sampling these independent anatomical entryways across the molar dentition, the eight-site framework effectively captures clinical variance from all four quadrants. Consequently, this balanced distribution ensures whole-mouth diagnostic reliability and widespread clinical utility.

A compelling biological justification for mandating this distributed 8-site grid emerged directly from post-hoc sensitivity analyses, which exposed severe localised blind spots when tracking isolated top-performing teeth instead of a more comprehensive protocol. In the derivation dataset, individual feature importance was primarily anchored by tooth 31, whereas in the external validation cohort, tooth 3 shifted to become the most dominant predictor (Supplementary Table S8). Had a tool been constructed by opportunistically picking only the top features of the training data, it would have lacked data on tooth 3 entirely—the primary predictor in the external validation set. Importantly, because this cross-cohort divergence was uncovered during subsequent sensitivity testing after the final model architecture had been fully locked, protection against inter-cohort variations was successfully achieved without risking data leakage or selection bias. This multi-rooted topology ensures true clinical transferability across diverse populations.

To further investigate the mathematical boundaries of this framework, an exploratory sensitivity analysis was executed utilising an expanded 16-surface configuration where all molars provided two predictive sites. While this more complex architecture achieved a higher absolute AUC of 0.863 (95% CI: 0.833–0.889)—representing an absolute increase of 0.004 over the primary framework—the parsimonious 8-site configuration was deliberately retained as the definitive screening tool. By standardising the screening protocol around this 8-site layout, a minor relative reduction in accuracy was intentionally traded for a profound 50% absolute reduction in chair-side data collection points. This structural economisation demonstrates that a single, strategically isolated surface per molar is fully sufficient to capture systemic disease variance without inflating clinical diagnostic workflows. Crucially, this tight convergence also serves as a mathematical safeguard against feature instability, as detailed later in the limitations.

This trade-off aligns seamlessly with modern dental informatics paradigms, particularly the concept of ‘Data Dentistry’ outlined by Schwendicke and Krois (2022) [39]. By integrating simplified, high-yield clinical inputs, this data-enriched framework echoes their call to improve the accessibility, affordability, and efficiency of specialised triage workflows. To facilitate seamless clinical adoption, this targeted documentation workflow can be further supported in real time through the integration of modern voice-assisted charting systems [40].

To maintain high diagnostic precision across international borders, local clinical implementation requires the empirical capture of population-specific molar probing depths to account for varying geographical disease phenotypes. Furthermore, it is critical that the specific molar teeth and their optimal probing depth sites are systematically re-examined and re-determined using the exact predictor-selection methodology established in this study. This empirical data capture facilitates essential algorithmic maintenance, allowing for the prospective recalibration of the baseline risk threshold and the precise fine-tuning of the model intercept to match local epidemiological realities.

Beyond these statistical adaptations, the parsimonious 8-site framework offers immediate structural compatibility with contemporary European and Nordic oral healthcare models, which heavily utilise dental hygienists as independent, frontline screening providers. Public clinic employment for dental hygienists stands at approximately 60% in Finland and 51% in Sweden [41]. In these public sectors, full-mouth clinical charting for every routine baseline visit is frequently precluded by tight scheduling constraints and financial resource limits. By functioning as a passive, automated electronic dental record filter that eliminates roughly 39 unnecessary dentist referrals per 100 individuals, the model delivers concrete public health utility. This positions the framework as a highly scalable operational asset capable of streamlining triage workflows and optimising resource allocation within public dental delivery systems outside the U.S. environment.

To maintain this epidemiological robustness within streamlined workflows, the underlying multivariable regressions explicitly account for the interdependency between active pocketing and historical tooth loss. By modelling site measurements and tooth presence indicators as coupled pairs (e.g., the C02A–T02 pair)—where binary presence indicators act as critical baseline predictors—the model successfully captures a crucial conditional contrast. Specifically, in a standard continuous predictor scale, a patient with a missing tooth—where the probing depth is coded as –1—would mathematically appear healthier than a patient with an ideal, healthy tooth with a probing depth of 0. To resolve this paradox, the separate binary tooth presence variable (where 0 indicates a missing tooth) introduces a structural adjustment into the regression equation. This allows the model to correctly interpret the –1 probing depth value as a missing tooth rather than a clinically superior state to a probing depth of 0. Failing to incorporate these conditional missingness architectures potentially introduces survivorship selection bias, as individuals with severe historical disease would appear artificially healthy if missing high-impact teeth were omitted from the scoring architecture.

The exclusion of traditional non-clinical risk factors—such as smoking, diabetes, socioeconomic status, oral hygiene, and previous periodontal treatments—was an a priori design decision, subsequently validated through post-hoc testing. The dependent variable utilises the NHANES ‘gum disease/problem’ status indicator as an epidemiological proxy for clinically relevant disease. To justify this parsimonious approach, smoking status, diabetes status, race/ethnicity, and education level were individually incorporated into the predictive framework. Each isolated addition yielded only marginal improvements in external validation discriminative power. Furthermore, Decision Curve Analysis at the 0.33 decision threshold demonstrated minimal variance in clinical utility. Specifically, the baseline model avoided 38.9 unnecessary comprehensive examinations by a dentist per 100 individuals, compared to 39.4 when the baseline was augmented with smoking, 39.0 with diabetes, 38.9 with ethnicity, and 39.2 with education.

Additionally, variables regarding oral hygiene habits and past periodontal treatments were intentionally omitted. Incorporating these parameters would not only severely compromise operational parsimony but also introduce significant validity issues, as self-reported questionnaire data regarding hygiene and periodontal treatment history within large-scale surveys are often subjective and highly susceptible to recall and social desirability biases. Furthermore, introducing questionnaire items that are conceptually too close to the predicted outcome risks introducing severe proximity and confounding biases, causing the algorithm to overfit on behavioural proximity rather than true underlying clinical risk patterns. From a biomedical informatics perspective, requiring such questionnaire inputs also introduces significant data-capture friction and missing data risks within automated EHR background software.

However, it can be concluded that these findings strongly indicate a biological redundancy within the framework; tobacco-induced tissue destruction, systemic diseases, and socioeconomic disparities are effectively captured and manifested by the intraoral clinical variables alongside age and gender. Despite the established epidemiological merit and clinical utility of these behavioural and systemic metrics, the current framework prioritises minimal data-capture friction to guarantee seamless initial deployment, leaving the exact quantification of their incremental clinical value to be explored in future studies.

Looking beyond electronic health record metrics, a parallel strategy for capturing active disease dynamics involves integrating molecular-level inflammatory signals. Leveraging readily available point-of-care biomarkers, the analysis of active matrix metalloproteinase-8 (aMMP-8) from mouthrinses represents a highly viable modality to augment gum disease detection, as varying biomarker concentrations are associated with both the presence and severity of periodontitis [42]. This biomarker offers substantial utility for patient-centred monitoring and screening initiatives, introducing new dimensions to predictive risk stratification. When deployed in institutional settings, point-of-care aMMP-8 tests paired with digital readers at the dental clinic equip clinicians with objective, high-resolution metrics to monitor disease dynamics [43]. Such technological synergy complements routine workflows. By integrating these immediate, non-invasive biomarker results directly into clinic records, the algorithm provides an instant, data-driven baseline for clinical decision-making.

Whether deployed as a standalone software framework or augmented by such molecular diagnostics, this type of objective, algorithmic support is particularly valuable for accelerating clinical decision-making among allied dental professionals. This alignment is highly supported by global dental public health frameworks, including the World Health Organization (WHO) and The Lancet Series on Oral Health, which strongly advocate for expanding the clinical scope of frontline dental practitioners to address population-level disease burdens [44,45]. Recent pan-European data underscores that legal and structural frameworks are actively evolving to grant dental hygienists greater clinical autonomy, including the reduction in direct dentist supervision and the expansion of independent practice capabilities in countries like the Netherlands and Denmark [41]. Providing these frontline screening experts with a validated, software-driven triage tool allows them to confidently risk-stratify patients during initial assessments, thereby reinforcing their expanding role in optimising patient care pathways and clinical workflows within modern oral healthcare teams.

Several study limitations warrant structured consideration. A primary methodological concern involves the inherent risk of selection optimism and data-driven bias during backward elimination loops when modelling a 34-variable exploratory pool under sample compression. In this study, the exploratory Phase 1 feature selection phase was restricted to a highly filtered complete-case training subset (Figure 2). At the exact entry point of the selection algorithm—when the initial probing depth parameters were evaluated—the analysis operated under a highly compressed unweighted events-per-variable (EPV) ratio of 7.8. This low baseline floor inherently increased the structural susceptibility to overfitting and sub-optimal variable isolation.

This potential selection vulnerability was formally cross-examined from a technical perspective via the post-hoc sensitivity analysis. Cross-cohort evaluation confirmed that any potential performance decrement stemming from the primary framework was not substantial; the 8-site configuration yielded an external validation AUC of 0.859, compared to 0.863 for the expanded, saturated 16-surface architecture (a shift in merely 0.004). This tight convergence confirms that even if marginal feature instability was introduced by the baseline EPV floor, the isolated 8-site configuration captured the bulk of available whole-mouth disease burden. From a methodological standpoint, this rendering of alternative predictor combinations as clinically redundant statistically justifies the retention of the parsimonious model.

In tandem with this reduction, model stability improved dramatically during the final stages of the feature selection loop. Just before the 10 core predictors (the eight selected probing depth sites, age, and gender) were locked, the unweighted EPV ratio rose to a stable 26.4. This threshold represents an acceptable level of numerical consistency and robust parameter estimation according to established methodological standards [30]. Transitioning back to the full dataset for Phase 2 expanded the sample size under the design-adjusted complex samples framework, safely consolidating the final feature density to a highly robust EPV ratio of 40.7 across the final 18 parameters. Because this transition and the subsequent independent external validation sustained a high discriminative capacity (pooled external validation AUC = 0.859), any mathematical instability or data-driven bias stemming from the initial EPV floors can be considered minimised, safeguarding the integrity and generalisability of the final screening tool.

Although the restriction to eight specific molar sites was robustly justified by statistical constraints and clinical efficiency, alternate tooth types and configuration strategies were not exhaustively explored. Future multi-faceted epidemiological investigations are warranted to systematically analyse different teeth, morphological groups, and localised surface combinations within the NHANES cycles. Such dedicated future research will be essential to evaluate whether alternative parsimonious configurations can further optimise diagnostic precision and maximise screening utility across diverse clinical workflows.

Beyond these statistical and algorithmic factors, direct causal inferences are fundamentally restricted by the cross-sectional architecture of consecutive NHANES cohorts; consequently, a contemporaneous point-in-time risk profile is reflected by the generated screening output rather than an epidemiological prediction of future longitudinal disease incidence. Another distinct constraint stems from the target age spectrum; because the study framework was explicitly bounded within the 30–50-year demographic to capture the pivotal early therapeutic window, the current algorithm’s performance parameters cannot be extrapolated to older populations exhibiting significantly higher systemic tooth loss without fundamental mathematical remodelling.

To build on these foundational findings, the robust performance demonstrated through independent external temporal validation establishes the essential prerequisite for live clinical deployment. The next logical operational phase requires prospective clinical validation within real-world, high-throughput dental and primary care networks. These future implementation studies will be valuable to quantify live user experience, evaluate automated data-capture latency, and monitor longitudinal referral compliance in diverse clinical workflows. This line of research is necessary to monitor long-term patient flows, track actual referral-to-treatment intervals, and reliably project subsequent dental care resource demands [46].

To sustain high levels of diagnostic performance over time, proactive algorithmic maintenance—including localised periodic fine-tuning—must be routinely executed to ensure the framework remains fully optimised across diverse clinical and public health contexts. Finally, a strict commitment to robust institutional oversight and ethical data governance must be maintained during software deployment to guarantee continuous, uncompromised adherence to all regional legal frameworks, patient confidentiality parameters, and electronic health record privacy requirements. Ultimately, the robust, tightly bounded AUC demonstrated during external validation strongly suggests that these acknowledged limitations did not critically impair the framework’s overall analytical performance or target population validity.

## 5. Conclusions

This study developed and externally validated a parsimonious screening framework that achieves high discriminative capacity and consistent generalisability for identifying “gum disease or related problem” status within the 30–50-year-old demographic. By incorporating an optimised eight-site probing depth protocol at the clinically prioritised threshold probability of (*Pt* = 0.33), Decision Curve Analysis substantiated a clear public health net benefit. By drastically reducing data collection points to minimise chairside time, this screening tool offers a validated, high-throughput pathway for seamless integration into electronic health records, primary care triage systems, and community-led public health programmes, enabling early diagnostic interception prior to irreversible alveolar bone destruction. Although prospective clinical implementation studies are warranted as the next operational phase to evaluate live workflow dynamics, automated data-capture latency, and longitudinal referral compliance, the high transportability and robust calibration sustained across independent national cohorts strongly support deploying this architecture as a scalable triage utility.

## Figures and Tables

**Table 1:** Baseline characteristics of variables included in the diagnostic modelling (*n* = 1732).

Characteristic	Analytic Sample (*n* = 1732)	Gum Disease/ Problem (36.7%)	No Gum Disease/ Problem (63.3%)
**Categorical Variables**	**Pooled (%)**	**Pooled (%)**	**Pooled (%)**
Gender			
Female	50.1%	37.3%	57.5%
Male	49.9%	62.7%	42.5%
**Binary Teeth Variables**	**Pooled (%)**	**Pooled (%)**	**Pooled (%)**
T02 present	87.2%	76.6%	93.3%
T03 present	87.2%	79.8%	91.5%
T14 present	86.1%	76.2%	91.9%
T15 present	86.6%	81.2%	89.7%
T18 present	85.4%	75.0%	91.4%
T19 present	79.7%	67.9%	86.6%
T30 present	81.1%	69.5%	87.7%
T31 present	86.0%	74.3%	92.7%
**Continuous Variables**	**Pooled Mean (SD)**	**Pooled Mean (SD)**	**Pooled Mean (SD)**
Age (yrs)	40.2 (6.2)	41.0 (5.9)	39.6 (6.3)
C02A	2.2 (0.9)	2.8 (1.0)	1.9 (0.7)
S03D	1.6 (0.9)	2.1 (1.0)	1.4 (0.6)
S14D	1.8 (1.0)	2.5 (1.1)	1.5 (0.7)
C15A	1.9 (0.9)	2.5 (1.0)	1.6 (0.6)
C18A	2.3 (0.9)	2.8 (1.0)	2.0 (0.7)
S19S	1.7 (0.8)	2.2 (0.9)	1.4 (0.7)
C30P	2.4 (1.1)	3.0 (1.2)	2.1 (0.8)
C31P	2.2 (1.0)	2.9 (1.1)	1.9 (0.8)

Note: Values represent descriptive baseline statistics (means with SD or population-weighted percentages) pooled across 20 multiple-imputation cycles (*n* = 1732). To account for the complex NHANES design, analyses incorporated stratification, clustering, and mobile examination centre weights (WTMEC2YR). Alphanumeric codes indicate site-specific measurements (mm): prefix denotes surface (S = buccal, C = lingual), numbers specify tooth position, and final character indicates orientation (D/P = distal, S/A = mesial); e.g., C02A is the lingual-mesial site of tooth 02. Structural missing codes (−1) representing missing teeth were excluded from continuous variables (C02A–C31P), meaning that the reported continuous means represent present teeth only. Full baseline profiles, including socio-demographic and lifestyle characteristics, are detailed in Supplementary Table S7. PD values range from 0 to 5 mm, with all measurements exceeding 5 mm truncated to 5 mm.

**Table 2:** Multivariable logistic regression analysis of variables for predicting individual’s “gum disease/problem” status (Training Set, *n* = 1732, NHANES cycle 2011–2012).

Predictor Variable	B	S.E.	aOR (95% CI)	*p*
C02A	0.355	0.172	1.426 (1.017–1.999)	0.044
S03D	0.29	0.162	1.336 (0.972–1.837)	0.077
S14D	0.448	0.165	1.566 (1.129–2.171)	0.008
C15A	0.721	0.206	2.056 (1.369–3.087)	<0.001
C18A	0.553	0.157	1.738 (1.278–2.376)	<0.001
S19S	0.599	0.165	1.821 (1.315–2.521)	<0.001
C30P	0.282	0.201	1.326 (0.895–1.966)	0.166
C31P	0.553	0.131	1.739 (1.346–2.248)	<0.001
T02 (absent = 0, present = 1)	−1.886	0.588	0.152 (0.048–0.480)	0.002
T03 (absent = 0, present = 1)	−0.666	0.459	0.514 (0.209–1.262)	0.149
T14 (absent = 0, present = 1)	−2.173	0.56	0.114 (0.038–0.341)	<0.001
T15 (absent = 0, present = 1)	−1.863	0.696	0.155 (0.040–0.607)	0.008
T18 (absent = 0, present = 1)	−2.133	0.611	0.118 (0.036–0.392)	<0.001
T19 (absent = 0, present = 1)	−2.371	0.539	0.093 (0.033–0.269)	<0.001
T30 (absent = 0, present = 1)	−1.012	0.679	0.364 (0.096–1.475)	0.141
T31 (absent = 0, present = 1)	−2.377	0.544	0.093 (0.032–0.270)	<0.001
Gender (female = 0, male = 1)	0.741	0.173	2.098 (1.495–2.945)	<0.001
Age (years)	0.033	0.014	1.034 (1.006–1.062)	0.015
Constant	3.793	0.828	–	<0.001

Note: Estimates were pooled across 20 multiple-imputation datasets using Rubin’s rules. Analyses accounted for the complex NHANES design. Age and probing depth (PD) metrics were modelled as continuous variables; reference categories are “Absent” (0) for tooth presence and female (0) for gender. Statistical estimates represent the fully adjusted multivariable framework; crude univariable results are provided in Supplementary Table S9. To account for missing teeth (coded as −1 in PD data), continuous PD models were adjusted for tooth presence and vice versa. Model fit: Pooled training Nagelkerke Pseudo-R^2^ = 0.593.

## Data Availability

The datasets analysed during the current study are available in the NHANES repository.

## Supplementary Materials

The following supporting information can be downloaded at: https://www.mdpi.com/article/10.3390/dj14090560/s1: Figure S1: Prevalence of the dependent variable (includes Supplementary Table S1: Weighted 95% confidence intervals (CI) corresponding to the prevalence rates shown in Supplementary Figure S1); Note S1: User interaction of the model; Note S2: Robustness of the dependent variable (includes Supplementary Table S2: Diagnostic performance of clinical attachment level (CAL) thresholds in predicting gum disease/problem); Note S3: Non-response analysis (includes Supplementary Table S3: Survey-adjusted baseline comparison between the excluded partition (*n* = 387) and the retained sensitivity analysis cohort (*n* = 3978) derived from the initial age-eligible framework (Total baseline *n* = 4365)); Note S4: Multiple imputation; Note S5: Predictor Selection; Note S6: Handling missing inputs; Table S4: Missing data patterns (*n* = 3605); Table S5: Missing data patterns (*n* = 1732); Table S6: Coding of multivariable model predictors; Note S7: Results of model updating; Note S8: Prediction model specification and access; Note S9: Cut-off selection for the prediction model; Table S7: Expanded characteristics; Table S8: Feature impacts on model performance; Table S9: Univariable and bivariable models; Table S10: Complete-Case vs. imputed Model; Table S11: External regression analysis; Note S10: Estimating the threshold for DCA; Note S11: Methodological sensitivity analysis.
