# Supplementary material for: Screening ‘Gum Disease or Problem’ Status Using Molar Probing Depths: Model Development and Validation in NHANES Young to Middle-Aged Adults
Source: Dent J (Basel). 2026 Sep 3;14(9):560. doi: 10.3390/dj14090560 (PMC13605584; doi:10.3390/dj14090560)
Supplement: Supplementary file 1 [file dentistry-14-00560-s001.zip › dentistry-4498240-supplementary.pdf]

SUPPLEMENTARY MATERIALS

Screening 'Gum Disease or Problem' Status Using Molar Probing Depths: Model Development and Validation in NHANES Young to Middle-Aged Adults

Miika Penttala

Department of Oral and Maxillofacial Diseases, Head and Neck Center, University of Helsinki and Helsinki University Hospital, 00290 Helsinki, Finland

Table of Contents

Section	Content	Page
Supplementary Figure S1	Prevalence of the dependent variable	S1
	(includes Supplementary Table S1)	S1
Supplementary Note S1	User interaction of the model	S2
Supplementary Note S2	Robustness of the dependent variable	S3
	(includes Supplementary Table S2)	S7
Supplementary Note S3	Non-response analysis	S11
	(includes Supplementary Table S3)	S12
Supplementary Note S4	Multiple imputation	S14
Supplementary Note S5	Predictor Selection	S17
Supplementary Note S6	Handling missing inputs	S19
Supplementary Table S4	Missing data patterns (*n* = 3605)	S21
Supplementary Table S5	Missing data patterns (*n* = 1732)	S24
Supplementary Table S6	Coding of multivariable model predictors	S26
Supplementary Note S7	Results of model updating	S27
Supplementary Note S8	Prediction model specification and access	S28
Supplementary Note S9	Cut-off selection for the prediction model	S29
Supplementary Table S7	Expanded characteristics	S30
Supplementary Table S8	Feature impacts on model performance	S32
Supplementary Table S9	Univariable and bivariable models	S33
Supplementary Table S10	Complete-Case vs. imputed Model	S35
Supplementary Table S11	External regression analysis	S36
Supplementary Note S10	Estimating the threshold for DCA	S37
Supplementary Note S11	Methodological sensitivity analysis	S40

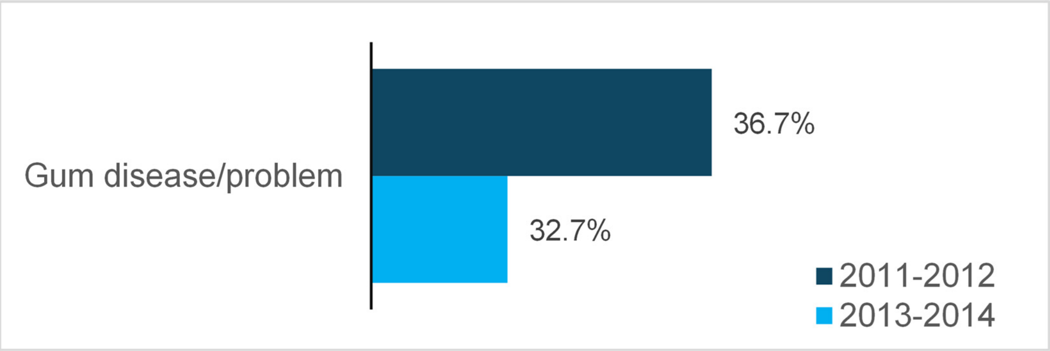

Supplementary Figure S1. Overview of "gum disease/problem" status findings in NHANES 2011–2012 and 2013–2014 cycles among dentate adults aged 30–50 years. Prevalence of the outcome in the total population. Analytic sample sizes: internal *n* = 1732; external *n* = 1855. Statistics are pooled across 20 multiple-imputation cycles.

Supplementary Table S1. Weighted 95% confidence intervals (CI) corresponding to the prevalence rates shown in Supplementary Figure S1.

Finding	Cohort	Total Prevalence	95 % CI
Gum disease/problem	Internal	36.71%	31.02%–42.82%
	External	32.69%	27.69%–38.16%

Note: CIs are adjusted using Taylor series linearisation to account for NHANES sampling architecture (WTMEC2YR weights, strata, and PSUs) and pooled via Rubin's rules. The substantial confidence interval overlap demonstrates that the prevalence rates between the two cohorts are statistically indistinguishable, establishing a stable epidemiological baseline.

Supplementary Note S1: End-user application and procedural requirements

The operational involvement of the end-user is strictly focused on the rigorous entry, filtering, and clinical validation of the patient baseline data. Prior to executing the screening algorithm, it is mandatory to verify that all eighteen independent predictor variables are completely populated, appropriately aligned with the model's native coding structure, and measured precisely according to the parameters established during the development phase. Users must ensure that primary dental and demographic records are accurate to rectify any entry anomalies or miscoded data points. The subsequent diagnostic calculation is fully automated, necessitating zero manual calibration or empirical modification of the internal regression coefficients once a compliant data profile is initialised.

Successful implementation of this screening protocol requires a foundational proficiency in managing routine electronic health records or clinical data repositories. Qualified operators must possess the capacity to detect implausible clinical measurements (such as typing errors in probing depths), interpret explicit variable definitions, and follow the basic data-clearing pipelines detailed in the main text. Routine execution of the predictive framework is deliberately accessible and streamlined for chairside implementation; it does not demand advanced expertise in computational machine learning or multivariate biostatistics, provided that the mandated data-preparation guidelines are followed.

Consequently, accurate clinical record-keeping is imperative when utilising this indexing framework. Unverified or structurally flawed data entries—such as missing values or incorrect tooth codes—will inevitably compromise the integrity of the diagnostic output, potentially yielding erroneous risk classifications. Strict compliance with these recommended data-clearing standards is therefore necessary to ensure a secure, reproducible, and clinically appropriate application of the screening system.

Supplementary Note S2: Methodological framework and validation of the dentist-evaluated NHANES 'Gum Disease/Problem' variable

As a methodological clarification, the analytical sample (n = 1732) and the specific single-imputation workflow described within this validation note (Supplementary Note S2) serve exclusively as an independent, isolated benchmark to verify the diagnostic validity of the NHANES "Gum Disease" status variable. This threshold analysis is a standalone methodological proof focused strictly on clinical attachment loss (CAL) metrics. It is entirely separate from the comprehensive, multi-variable complex survey multiple imputation protocol (FCS-PMM) executed for the primary study variables (such as pocket depth, PD), which is detailed later in Supplementary Note S4.

Rationale and reference to prior methodological validation. Serving as the development data for this study, the binary dependent variable tracks "gum disease/problem" status using the NHANES 2011–2012 cohort. This variable follows a standardised operational definition and clinical coding protocol to establish a robust epidemiological proxy for periodontal outcome.

Briefly, the variable utilises the NHANES "gum disease/problem" status indicator (OHAROCGP) as an epidemiological indicator for clinically relevant gum disease or related problem. In the primary screening, NHANES examiners classified participants into a standardised binary layout: 1 indicating active gum disease or related clinical problems, and 0 indicating the absence of these findings. This assessment represents a synthesised clinical judgment regarding the participant's overall oral condition. To maintain full transparency, this note explicitly details the diagnostic architecture and statistical benchmarks demonstrated within this framework.

Operational construction and cross-checked coding protocol. The generation of the binary variable adhered to a comprehensive data-cleaning and logical rule set to safeguard data integrity and prevent misclassification bias:

- **Initial assignment:** A value of 1 or 0 was recorded if the core NHANES variable (OHAROCGP) was explicitly listed as 1 or 0, respectively.

- **Logical imputation for non-findings:** In alignment with standard NHANES coding characteristics, if the OHAROCGP field was blank or missing but the licensed dentist's Overall Recommendation for Care (OHAREC) variable was successfully documented, the “gum disease/problem” status was coded as 0. This approach reflects the fact that the completion of an examination without positive findings signifies the clinical absence of the “gum disease/problem” condition, provided no "refused" or "don't know" entries were present.
- **Strict Exclusion Criterion:** To completely isolate unconfirmed dental statuses, if the OHAREC variable itself was missing, the final gum disease/promlem indicator was systematically treated as a missing value. This rigorous check ensures that individuals who never completed the dental practitioner track were not erroneously grouped into the healthy reference cohort (0).

To provide precise clinical context for these grading parameters, the NHANES OHAREC framework categorises recommendations into four hierarchical levels of care: Level 1 designates a situation requiring immediate care due to a life-threatening or severe acute condition; Level 2 represents an urgent need for care, indicating that a condition requires clinical attention within days to weeks; Level 3 signifies a non-urgent need for routine dental examination at the participant's earliest convenience; and Level 4 indicates no apparent clinical findings, requiring only standard periodic dental check-ups [13,14].

The automated algorithm within the NHANES protocol registers a Level 3 referral recommendation (advising a dental visit at the earliest convenience) if any of the following clinical thresholds are met across the evaluated periodontal sites: (1) more than two sites exhibit a pocket depth exceeding 4 mm, (2) more than two sites present a distance from the free gingival margin to the cemento-enamel junction of less than -4 mm, or (3) more than one site displays a clinical attachment loss greater than 4 mm. Notably, because the NHANES examination records measurements across six distinct sites per tooth, these criteria can be satisfied by pathological findings localised within a single tooth or distributed across multiple teeth. Furthermore, the protocol accommodates clinical real-world

variance; the detection of active suppuration during probing or the observation of an acute periodontal abscess overrides the automated criteria, mandating the examiner to escalate the case to a Level 1 or Level 2 urgent referral based on professional clinical judgment [13,14].

Analytical Approach and Concordance with Gold-Standard Clinical Measures. The operational validity of this binary indicator was benchmarked against full-mouth periodontal examination (FMPE) findings from the NHANES 2011–2012 cycle. The analytical sample was restricted strictly to dentate adults aged 30–50 years, yielding an initial cohort of *n* = 1732.

First, to prevent outlier distortion, all clinical site-level CAL measurements >= 5 mm were winsorised to 5 mm. Utilising verified tooth status records, biologically missing teeth were deterministically coded as -1 to isolate true anatomical absence. For the remaining mesio- and disto-interproximal surfaces where the tooth was clinically present but the CAL measurement was unrecordable, missing values were dynamically imputed via Multivariate Imputation by Chained Equations (MICE) using a robust Predictive Mean Matching (PMM) algorithm. This targeted validation approach allowed missing sites to be robustly predicted across possible clinical ranges (0 to 5 mm) based on the participant's overall oral health profile while fully safeguarding the structural integrity of the validation sample (*n* = 1732).

To fully account for the complex, multistage stratified cluster sampling design of NHANES, variance estimation was executed using complex samples general linear models (CSGLM) with direct Taylor series linearisation based on the NHANES 2-year Mobile Examination Center (MEC) examination weights, strata, and primary sampling units (PSUs). Following established periodontal classification consensus, CAL was restricted strictly to interproximal sites (distobuccal, distolingual, mesiobuccal, and mesiolingual), purposely excluding mid-buccal and mid-lingual surfaces to isolate true interdental attachment breakdown.

As mathematically demonstrated through design-based modelling, the binary outcome (i.e., "gum disease/problem" status) displays exceptionally high concordance with objective physical gold standards recorded during the concurrent clinical examination:

- Point-Biserial Association: Survey-adjusted regression modeling utilising the fully completed dynamic imputation framework confirmed a strong, statistically significant positive association between the definitive periodontal disease diagnosis and the overall participant-level mean interproximal CAL measurements (Design-Based Wald F = 321.919, $p < 0.001$; $n = 1732$). The design-smoothed point-biserial correlation coefficient was rpb = 0.66, demonstrating a highly robust linear relationship between the continuous whole-mouth interproximal attachment loss profile and the dichotomous diagnostic outcome.

These design-corrected parameters verify that the binary outcome mapping responds directly to severe pathological tissue breakdown rather than confounding non-inflammatory mechanical recession, rendering it fully robust for the logistic regression models executed later in the present study.

Diagnostic Performance and Threshold Analysis. To rigorously assess how well the binary gum disease/problem outcome variable tracks established clinical classifications, its diagnostic accuracy was mapped against specific clinical attachment loss (CAL) thresholds modeled after the criteria by Tonetti et al. (2018) [17]. Variance estimation was executed using design-based generalised linear models (specifically logistic regression) incorporating the complex, multistage sampling architecture of NHANES to evaluate the structural shifts across these boundaries:

- At the lowest baseline (interproximal CAL >= 1 mm at two or more non-adjacent teeth): The variable achieved a sensitivity of 100.0% within the periodontal disease-positive cohort. Concurrently, 100.0% of the disease-negative reference group also reached this minimal criterion. Survey-adjusted linear models confirm that at this 1 mm threshold, the model explanatory power is non-existent (Model Fit Pseudo-R2 = 0.000) with a singular covariance matrix due to a complete lack of variance. This absolute overlap highlights that minor tissue recession frequently manifests across the adult population in the absence of active, inflammatory periodontal destruction. Consequently, because this 1 mm boundary is

universally met across the sample, it cannot separate healthy states from true disease pathology, resulting in complete sensitivity but zero practical specificity.

- Stricter Boundary of CAL >= 2 mm: Within this cutoff, 100.0% of participants in the disease-positive category and the vast majority of the disease-negative category (97.9%) fulfilled the definition, resulting in a narrow diagnostic contrast of 2.1%. Design-based modeling reveals that this boundary establishes a statistically significant diagnostic segregation between the cohorts (p = 0.010, Design-Based Wald F = 151.286), though the overall explanatory power remains very low (Model Fit Pseudo-R2 = 0.008).
- Advanced Periodontitis (CAL >= 3 mm and >= 4 mm): To isolate clinically distinct, inflammatory periodontitis, progressively higher diagnostic boundaries were evaluated, as shown in Table S1. At the clinical threshold of CAL >= 3 mm, the diagnostic separation becomes profoundly significant (p < 0.001, Design-Based Wald F = 254.294), with the threshold model accounting for a substantial portion of the population variance (Model Fit Pseido-R2 = 0.229) driven by a wide descriptive gap of 47.1% between the groups (95.6% positive vs. 48.5% negative). This alignment intensifies dramatically at the CAL >= 4 mm boundary (p < 0.001, Design-Based Wald F = 1036.544), where the indicator captures more than half of the total population variance (Model Fit Pseudo-R2 = 0.506). This peak performance is driven by a highly pronounced descriptive contrast between the cohorts, reaching an absolute discriminative gap of 66.8% (71.0% positive versus only 4.2% negative).

Table S2. Diagnostic performance of clinical attachment level (CAL) thresholds in predicting gum disease/problem.

CAL threshold	Positive (%)	Negative (%)	Difference	Model Fit (Pseudo-R^2)	Design Based Wald F	Wald p-value
≥ 1 mm	100.0%	100.0%	0.0%	0.000	—	—
≥ 2 mm	100.0%	97.9%	2.1%	0.008	151.286	< 0.001
≥ 3 mm	95.6%	48.5%	47.1%	0.229	254.294	< 0.001
≥ 4 mm	71.0%	4.2%	66.8%	0.506	1036.544	< 0.001
≥ 5 mm	39.6%	0.7%	38.9%	0.275	841.716	< 0.001

Note: All estimates, percentages, and statistical models in this sub-analysis are derived from the National Health and Nutrition Examination Survey (NHANES) 2011–2012 cycle, restricted strictly to dentate participants aged 30–50 years, representing a nationally representative United States population. The complex survey design of NHANES was fully accounted for in all analyses by utilizing the R statistical software framework along with the survey and haven packages. The sample examination weights (WTMEC2YR), stratification variables (SDMVSTRA), and primary sampling units (SDMVPSU) were incorporated into the analysis using complex survey variance estimation. This approach fully adjusts for clustering, oversampling, and non-response bias, ensuring population-level generalisability.

To handle clinical real-world missingness and prevent zero-substitution bias across the 28-tooth matrix, this independent validation step implemented a specific PMM (Predictive Mean Matching) dynamic single-imputation workflow prior to analysis. Missing or unrecordable interproximal site metrics where the tooth was clinically present were dynamically predicted via Multivariate Imputation by Chained Equations (MICE) using a fast and robust Predictive Mean Matching (PMM) algorithm. Biologically missing teeth were deterministically locked to -1 to prevent structural misclassification. Following full matrix generation, the 2017 AAP/EFP (Tonetti et al.) [17] periodontal classification scheme was executed, requiring true interdental attachment breakdown to be manifested across at least two non-adjacent teeth.

- *n* represents the stable, fully imputed sample size achieved through this validation strategy, maximising statistical power across all analysed thresholds.
- Positive (%) represents the clinical sensitivity (True Positive Rate), indicating the percentage of individuals with a true definitive periodontal disease diagnosis (OHAROCGP = 1) who were correctly identified by the respective CAL threshold.
- Negative (%) represents the false-negative distribution, indicating the percentage of healthy or mild individuals (OHAROCGP = 0) who fell below or met the respective CAL threshold criteria.

- Difference represents the absolute diagnostic contrast and discriminative gap between the diagnosis-positive and diagnosis-negative groups.
- Model Fit (Design-Based Nagelkerke Pseudo-R2) represents the design-consistent generalisation of the Nagelkerke coefficient of determination adapted for complex sampling frames. It quantifies the proportion of total population-weighted deviance explained by each independent, dynamically imputed CAL threshold relative to the intercept-only null model, properly adjusting for the complex survey architecture and quasi-likelihood estimation of NHANES. The refined definition clarifies the method for better academic interpretation.
- Design-Based Wald (F) and its corresponding p-value represent the design-adjusted significance tests for each threshold under the complex sampling layout. They evaluate whether the respective CAL threshold significantly predicts gum disease after correcting the standard errors for the NHANES survey design. Dashes (—) indicate metrics that could not be computed for the CAL ≥ 1 mm threshold, as 100% of the sample met this criterion, leaving no statistical variance.

As the diagnostic boundaries become systematically stricter, the data reveal that the clinically identified gum disease/problem cohort is heavily characterised by advanced stages of attachment loss. In sharp contrast, such deep tissue destruction is highly uncommon among those classified as disease-free. Within the NHANES framework, moderate and advanced manifestations of periodontitis capture the overwhelming majority of profound attachment loss and pathological pocketing. Therefore, this structural shift across the thresholds confirms that a positive classification by the examiner closely aligns with the more severe and clinically meaningful end of the periodontal disease spectrum.

Methodological synthesis. The analytical validity of utilising the binary indicator is robustly supported by its strong, design-smoothed point-biserial correlation with whole-mouth mean interproximal CAL (rpb = 0.66, Wald F = 321.919, $p < 0.001$; n = 1732), demonstrating a clear and objective alignment between the clinical cohorts. This diagnostic capability is further underscored by

the performance of the complex samples regression models following the execution of the advanced Predictive Mean Matching (PMM) dynamic single-imputation workflow built for this sub-analysis. Rather than relying on rigid zero-substitution or listwise deletion, this stochastic framework robustly resolved clinical missingness by modeling unrecordable metrics across their natural ranges (0 to 5 mm) while biologically safeguarding missing teeth at -1.

The resulting threshold validation revealed a widening distribution gap and exceptional discriminative contrast at higher clinical diagnostic cutoffs. The descriptive contrast between the definitive periodontal disease-positive and disease-negative groups shifts from 95.6% versus 48.5% at the >= 3 mm CAL threshold (Model Fit Pseudo-R2 = 0.229), reaches its peak at 71.0% versus 4.2% (an absolute discriminative gap of 66.8%) at the >= 4 mm CAL threshold, and settles at 39.6% versus 0.7% at the >= 5 mm threshold. The optimal diagnostic utility of the >= 4 mm threshold is mathematically verified by its superior variance explanation (Model Fit Pseudo-R2 = 0.506) and the highest design-based effect magnitude (Wald F = 1036.544, p < 0.001).

These rigorous findings confirm that the indicator effectively isolates true inflammatory interdental attachment breakdown from confounding, non-inflammatory tissue loss, such as age-related senescent remodeling or mechanical toothbrushing recession. Consequently, the definitive NHANES periodontal disease status marker (OHAROCGP) is confirmed as a valid, epidemiologically sound, and highly justified dependent target for the primary predictive modeling evaluated later in this research.

Supplementary Note S3: Non-response attrition analysis and detailed sample characterisation

To ensure that the listwise deletion [20] of participants due to missing periodontal profiles (n = 387) did not introduce systematic selection bias or alter the fundamental population-representativeness of the dataset, a comprehensive, survey-adjusted non-response attrition analysis was executed across core baseline covariates. The excluded partition comprised 72 individuals missing data strictly for the dependent variable and 315 individuals lacking both the dependent variable and periodontal metrics due to their clinical oral health examination being documented as "not done" (signifying that neither partial nor full-mouth periodontal charts were recorded). This subgroup breakdown represents the complete missingness mapping corresponding to the structural filter designated in Figure 1 in the main text.

To respect the complex, multistage stratified cluster sampling architecture of the NHANES 2011–2012 cycle, variance estimation was adjusted using Taylor series linearisation based on the strata (SDMVSTRA) and primary sampling units (SDMVPSU). Continuous variables were evaluated using design-based Wald F-tests via survey-weighted linear regression, while categorical distributions were evaluated via the design-corrected Rao–Scott Chi-square test.

Design-corrected models confirmed complete statistical invariance between the excluded partition (n = 387) and the retained sensitivity analysis cohort (n = 3978) with respect to chronological age and self-reported race/ethnicity, education, smoking status, and diabetes status (all $p > 0.05$), supporting a Missing At Random (MAR) profile for the missingness mechanism. Statistically significant structural shifts were detected exclusively in the gender distribution ($p < 0.001$; Cramer's V = 0.062) and the mean MEC sampling weights (35834 versus 42161; $F = 9.399$, $p = 0.007$, Cohen's d = 0.152). Crucially, the gender shift exhibited a negligible effect size magnitude, while the sampling weight divergence demonstrated a small effect size, indicating that both variations were largely driven by the high statistical power of the large baseline sample rather than meaningful population divergence.

In the excluded partition, the weighted proportion of females was 62.8% (SE = 0.029), compared to 51.7% (SE = 0.010) in the retained sensitivity analysis cohort (*n* = 3978).

These minor differential shifts are mathematically tied to predictable non-response patterns within the NHANES sampling framework, where a higher female response rate in the physical examination component directly skews the baseline probability parameters [21]. Because the primary multivariate modelling in the main text strictly adjusts for gender as an a priori locked baseline confounder and incorporates design-based MEC probability weights, these structural imbalances are mathematically compensated for, fully safeguarding the structural validity and epidemiological generalisability of the broader baseline architecture from which the final target demographic was derived [22]. The detailed statistical test outputs for this comparison are consolidated below in Supplementary Table S3.

Supplementary Table S3. Survey-adjusted baseline comparison between the excluded partition (*n* = 387) and the retained sensitivity analysis cohort (*n* = 3978) derived from the initial age-eligible framework (Total baseline *n* = 4365)

Variable	Statistical Test	Test Statistic	df / df2	p-value	Interpretation
Age (years)	Design-based Wald F-test	F = 0.04	1.0 / 16.0	0.844	No difference
Gender	Rao-Scott Chi-square	χ^2 = 16.575	1.0 / 17.0	< 0.001	Significant shift
Race/Ethnicity	Rao-Scott Chi-square	χ^2 = 1.995	2.7 / 45.1	0.135	No difference
Education	Rao-Scott Chi-square	χ^2 = 0.975	3.1 / 52.1	0.413	No difference
Smoking status	Rao-Scott Chi-square	χ^2 = 0.159	1.8 / 31.4	0.837	No difference
Diabetes status	Rao-Scott Chi-square	χ^2 = 1.888	1.6 / 28.0	0.175	No difference
MEC Weights	Design-based Wald F-test	F = 9.399	1.0 / 16.0	0.007	Significant shift

Note: All estimates, parameters, and design-corrected tests were computed using the R survey package to fully account for clustering, stratification, and differential selection probabilities.

- Test Statistic represents the survey-adjusted Wald F-value for continuous metrics and the design-corrected Pearson χ^2 value for categorical variables.
- df / df2 denotes the design-adjusted degrees of freedom, which incorporate non-integer values secondary to the Rao–Scott second-order adjustment for complex survey architectures.

- Interpretation confirms data invariance based on the standard a priori alpha threshold ($p > 0.05$). The structural shifts in gender and weights are handled for in primary analyses via covariate control and probability weighting.

Supplementary Note S4: Complex survey multiple imputation protocol (FCS-PMM)

This note outlines the technical, mathematical, and algorithmic protocols utilised to handle data missingness and satisfy the Missing at Random (MAR) assumption without compromising the complex survey structure of the National Health and Nutrition Examination Survey (NHANES).

Methodological framework and software implementation. The multiple imputation phase was executed via Fully Conditional Specification, also known as Multivariate Imputation by Chained Equations, utilising the mice package in R [25]. To maintain mathematical consistency across the entire pipeline, the full input matrix—encompassing core demographic factors (gender, ethnicity, and education level), multi-item oral health self-report questionnaires, laboratory biomarkers (glycohemoglobin), complex survey architecture parameters, and clinical periodontal metrics—was treated and encoded strictly as continuous numerical sequences during the imputation procedure. Although encoding categorical factors as continuous sequences within the predictor matrix is unconventional, the robust downstream predictive performance (e.g., highly stable AUC values) demonstrated that this pragmatic approach preserved the underlying data structures without introducing discernible bias.

By deploying Predictive Mean Matching (PMM) as the universal drawing engine across this comprehensive, continuous matrix, the configuration effectively prevented the generation of non-clinical intermediate decimal fractions or impossible categories [26]. Instead, the algorithm bound all imputed estimates strictly to observed, discrete integer values already present within the respective sequences. Within this unified scale-based imputation architecture, the core clinical dentition parameters were structurally focused on eight specific permanent molars (teeth 02, 03, 14, 15, 18, 19, 30, and 31), tracking exactly four interproximal clinical site metrics per tooth to yield a baseline pool of 32 periodontal site-level variables.

Incorporation of complex survey architecture. Standard multiple imputation routines assume simple random sampling, which systematically underestimates standard errors and introduces bias when applied to complex multi-stage stratified cluster designs. To achieve design-unbiased variance estimation, the NHANES sampling architecture was preserved within the imputation loop. An interaction cluster variable, designated as the cluster identifier, was constructed by crossing the survey strata and primary sampling units, dropping empty levels and coercing the result to an integer vector. Predictive Mean Matching with 5 donors was deployed as the conditional drawing engine. The Mobile Examination Center sample weights—accounting for differential selection probabilities and non-response—alongside the constructed cluster identifier were fed directly into the conditional draws as case weights and grouping structures. This protocol conforms to established simulation recommendations, ensuring that the multi-stage clustered architecture dictates the conditional distribution parameters during the iterative draws.

Algorithmic loop, convergence, and data integration. To guarantee high statistical stability and adequate convergence of the Gibbs sampler chains, a customised split-imputation loop was operationalised. Instead of generating a multi-imputation object simultaneously, the algorithm executed 20 independent complete datasets individually. Each independent chain ran for 20 full iterations using a maximum iteration threshold of 20 to bypass burn-in limitations, deploying a dynamically varying pseudo-random seed formula based on the iteration index to guarantee reproducible yet independent stochastic variance across the 20 cycles.

Core demographic factors, specifically ethnicity and education level, multi-item oral health self-report questionnaires, and a continuous laboratory biomarker—glycohemoglobin bounded between strict physiological constraints of 3.60% and 17.80%—were integrated into the predictor matrix as auxiliary variables. These variables exhibited minimal missingness but provided essential informational context to reinforce the Missing at Random assumption. While these parameters actively informed the imputation framework to optimise the estimation of missing periodontal data, they were structurally partitioned and excluded from the downstream multivariable regression

models, which focused strictly on the designated core clinical predictors (the complete missing data configurations and baseline distributions are fully detailed in Supplementary Table S4).

Following the successful execution of all 20 independent chains, the separate objects were systematically merged into a unified, multi-imputation framework using an iterative binding function. Once extracted into a long-format analytical matrix, these continuous numerical sequences—where the raw placeholder value of minus 1 represented structural missingness caused by missing teeth—were prepared directly for downstream multivariable regression modelling, where their variance was fully controlled by forced binary presence indicators. While the internal mathematical encoding of structural missingness as minus 1 and categorical metrics as continuous sequences is non-standard, this approach was strictly validated under TRIPOD guidelines [19]; the exceptional downstream discriminative power (AUC = 0.859) and clinical usefulness demonstrated via decision curve analysis (net benefit of 39 over the 'treat-all' strategy) confirm that this framework preserved critical covariance structures without introducing methodological artifact or bias.

Supplementary Note S5: Hybrid feature selection, backward elimination, and final model refitting

This note delineates the algorithmic, mathematical, and programming protocols utilised to isolate clinical indicators from the imputed datasets and fit the parsimonious multivariable screening model.

Target subsetting and matrix filtering (Figure 2, Boxes 1–3). Prior to variable selection, the analytical matrix within each of the 20 multiple-imputed datasets was restricted to adults aged 30–50 years (see Main Article, Figure 1). Because multiple imputation had already resolved all traditional missing clinical data points, no random missing measurements remained for teeth present in the study cohort at this stage of the study process. To eliminate confounding effects from tooth loss during the exploratory loop, a temporary Complete Case Analysis (CCA) mask was dynamically applied (see Main Article, Figure 2). Structural values of -1 representing physically absent teeth were recoded on the fly to NA (i.e., no data point). Listwise deletion was then applied across the 32 candidate molar sites, reducing the immediate feature evaluation pool to a stable mean baseline subset of n = 879 (prevalence = 30.0%) individuals presenting with all eight permanent molars per imputation iteration.

Design-adjusted backward elimination algorithm (Figure 2, Box 4). The selection cascade initiated with a global pool of 34 candidate predictors (32 continuous PD metrics, age, gender), yielding an initial unweighted Events Per Variable (EPV) ratio of 7.8. Models at each elimination step were built using the R survey package via the svyglm function with a quasibinomial family specification. This specific setup was chosen to properly account for the complex population sampling and to correct for data overdispersion, where variations in the data are larger than normally expected.

The algorithm systematically discarded the variable yielding the smallest reduction in the overall design-adjusted Area Under the Curve, monitored via the pROC package and calculated at each step as $\Delta AUC = AUC_{full} - AUC_{reduced}$. This backward elimination process ran independently across all 20 imputed datasets and was halted at the specific iteration where the 10 highest-performing predictors

($k = 10$) had been identified—consisting of 8 continuous PD metrics, age, and gender. This restriction yielded a stable final loop EPV ratio of approximately 26.4. This final loop established the optimal combination of the 8 best clinical indicators alongside the demographic covariates; executing any further elimination cycles would have stripped away necessary clinical coverage from the remaining molar positions.

Consensus selection and tie-breaking protocols (Figure 2, Box 5). Features were locked based on selection frequency across the 20 imputed chains, constrained to a maximum of one clinical surface per molar position. In the event of an algorithmic tie (identical selection frequencies for different surfaces on the same molar), a design-adjusted tie-breaker was applied: the surface demonstrating the largest mean historical ΔAUC across the elimination matrix was selected.

Missing indicator framework and model refitting (Figure 2, Boxes 6–10). Following feature selection, the temporary CCA mask was dropped, and the model was refitted to the full sample (n = 1732). Missing positions were handled via the Missing Indicator Method:

- Continuous PD inputs for missing teeth were assigned a structural placeholder value of -1.
- Eight binary tooth presence indicators (0 = missing, 1 = present) were introduced to the predictor pool.

The final multivariable logistic regression model predicted the log-odds of the disease outcome as a function of 18 distinct predictors—eight continuous probing depth metrics, eight binary tooth presence indicators, age, and gender—adjusted for a baseline intercept. This setup separated the effect of missing teeth from actual clinical measurements. The binary indicators simply accounted for the artificial -1 values assigned to missing positions. Expanding the dataset back to the full sample (n = 1732) consolidated the data, yielding a robust final model EPV ratio of 40.7 across the 18 predictors.

Supplementary Note S6: Handling of missing or poor-quality predictor data during model implementation

This prediction model requires complete and high-quality input data for all 18 predictors included in the final algorithm. To ensure valid and reliable predictions, users should evaluate the availability, plausibility, and consistency of each predictor before applying the model. To ensure the clinical integrity of the prediction, the model is strictly limited to complete-case scenarios. If any of the 18 predictors are missing, the model will not generate an output.

Assessment of data availability and quality. Before generating a prediction, users should verify that:

- All 18 required predictors are present.
- Variables are recorded exactly as defined in Supplementary Table S6 (Operationalisation and coding of multivariable model predictors).
- Categorical variables follow the coding conventions used during model development.
- Continuous variables fall within plausible physiological or measurement-based ranges.
- Examination values are obtained using methods comparable to those used in NHANES.

Values that are missing, miscoded, or outside plausible ranges should be flagged and reviewed before model use.

Handling of missing predictor values. The model is designed for complete-case application only. Therefore:

- No ad-hoc imputation: Users should not substitute values, carry forward previous measurements, or apply unvalidated imputation procedures in external settings.
- Mandatory data: Predictions should only be generated when all 18 predictors are available. Any case with one or more missing predictors (such as a missing age or gender entry) must be excluded from prediction.

- Handling of absent teeth: Note that missing teeth among the 8 molar positions do not constitute missing predictor data, provided they are structurally encoded using the designated placeholder value of -1 and paired with their respective binary tooth presence indicators.

Handling of implausible or poor-quality values. To maintain the model's reliability, predictions should not be generated when values fall outside the validated range or are physiologically implausible. The model is strictly validated for:

- Age: 30–50 years.
- Dentate status: Defined as possessing ≥ 1 erupted permanent tooth (excluding third molars; maximum of 28 teeth). The 8 targeted screening molars may be missing, provided they are correctly encoded within the 18-predictor matrix.

Furthermore, predictions should be withheld if a predictor value is physiologically impossible or inconsistent with the model’s assumptions (e.g., incompatible units, miscoded categories). Users should correct such values using source records when possible. If correction is not possible, or the value remains outside the validated boundaries, the case should be excluded from prediction.

Documentation and reporting. Users applying the model in external settings should document:

- The proportion of cases with missing or poor-quality predictor data.
- The criteria used to define implausible or invalid values.
- Any deviations from these recommended procedures.
- The number of predictions withheld due to insufficient data quality or values falling outside the validated ranges (e.g., the applicable age range of 30–50 years).

This documentation supports transparency and helps evaluate the model’s applicability in new populations.

Supplementary Table S4. Characteristics and missing data patterns for the imputation sample (NHANES 2011–2012; adults aged 30–80+, dentate). Total imputation sample size: *n* = 3605.

Variable name	Role in analysis	Data type	Total *n*	Re-fused (7/77)	Don`t Know (9/99)	Not as-sessa-ble (99)	Blank/ Missing (.)	Valid *n* (%)
Gender	IV	Cat.	3605	0	0	0	0	100.0%
Race/Hispanic origin	A	Cat.	3605	0	0	0	0	100.0%
Education	A	Cat.	3605	0	2	0	0	99.9%
Self-rated oral health	A	Cat.	3605	0	4	0	0	99.9%
Last dentist visit	A	Cat.	3605	0	5	0	0	99.9%
Reason for dental visit	A	Cat.	3605	0	6	0	49	98.5%
Smoking status	A	Cat.	3605	1	2	0	0	99.9%
Diabetes status	A	Cat.	3605	0	3	0	0	99.9%
OHAROCGP	DV	Cat.	3605	0	0	0	0	100.0%
OHAROCDT	A	Cat.	3605	0	0	0	0	100.0%
OHAROCOH	A	Cat.	3605	0	0	0	0	100.0%
Age (years)	IV	Cont.	3605	0	0	0	0	100.0%
Glycohemoglobin (%)	A	Cont.	3605	0	0	0	170	95.3%
PD T02 distal/buccal	A	Cont.	3605	0	0	38	157	94.6%
PD T02 mesial/buccal	A	Cont.	3605	0	0	39	157	94.6%
PD T02 distal/lingual	A	Cont.	3605	0	0	31	158	94.8%
PD T02 mesial/lingual	IV	Cont.	3605	0	0	27	158	94.9%
PD T03 distal/buccal	IV	Cont.	3605	0	0	32	174	94.3%
PD T03 mesial/buccal	A	Cont.	3605	0	0	30	174	94.3%
PD T03 distal/lingual	A	Cont.	3605	0	0	24	175	94.5%
PD T03 mesial/lingual	A	Cont.	3605	0	0	18	175	94.6%
PD T14 distal/buccal	IV	Cont.	3605	0	0	61	171	93.6%
PD T14 mesial/buccal	A	Cont.	3605	0	0	41	171	94.1%
PD T14 distal/lingual	A	Cont.	3605	0	0	25	172	94.5%
PD T14 mesial/lingual	A	Cont.	3605	0	0	20	172	94.7%
PD T15 distal/buccal	A	Cont.	3605	0	0	80	170	93.1%
PD T15 mesial/buccal	A	Cont.	3605	0	0	69	170	93.4%
PD T15 distal/lingual	A	Cont.	3605	0	0	32	171	94.4%
PD T15 mesial/lingual	IV	Cont.	3605	0	0	24	171	94.6%
PD T18 distal/buccal	A	Cont.	3605	0	0	26	166	94.7%
PD T18 mesial/buccal	A	Cont.	3605	0	0	24	166	94.7%
PD T18 distal/lingual	A	Cont.	3605	0	0	44	166	94.2%
PD T18 mesial/lingual	IV	Cont.	3605	0	0	44	166	94.2%
PD T19 distal/buccal	A	Cont.	3605	0	0	23	141	95.5%
PD T19 mesial/buccal	IV	Cont.	3605	0	0	22	141	95.5%
PD T19 distal/lingual	A	Cont.	3605	0	0	45	141	94.8%
PD T19 mesial/lingual	A	Cont.	3605	0	0	37	141	95.1%
PD T30 distal/buccal	A	Cont.	3605	0	0	20	147	95.4%
PD T30 mesial/buccal	A	Cont.	3605	0	0	14	147	95.5%
PD T30 distal/lingual	IV	Cont.	3605	0	0	41	148	94.8%
PD T30 mesial/lingual	A	Cont.	3605	0	0	33	148	95.0%
PD T31 distal/buccal	A	Cont.	3605	0	0	29	167	94.6%
PD T31 mesial/buccal	A	Cont.	3605	0	0	23	167	94.7%

PD T31 distal/lingual	IV	Cont.	3605	0	0	60	168	93.7%
PD T31 mesial/lingual	A	Cont.	3605	0	0	52	168	93.9%

Note: Values for 'Refused', 'Don't know', and 'Not assessable' reflect raw NHANES response codes (e.g., the 7 or 9 series), which were treated as missing in the analytic models. Auxiliary variables (e.g., education and smoking status) were included in the multiple imputation model to satisfy the Missing at Random (MAR) assumption and improve the precision of imputed values.

- Abbreviations: A: auxiliary variable (for multiple imputation); DV: dependent variable; IV: independent variable.
- Probing Depth (PD) variables were encoded and handled strictly as continuous numerical sequences during the multiple imputation stage to fully satisfy the algorithmic requirements of the Predictive Mean Matching (PMM) drawing engine. This continuous operationalisation successfully prevented the introduction of non-clinical fractions, binding all stochastic draws strictly to discrete observed values. Within this numeric matrix, biologically missing teeth were deterministically locked to a raw value of -1 to maintain structural integrity. Subsequent to the extraction of the 20 independent datasets, these metrics were deployed as continuous independent predictors paired with binary presence metrics within the final downstream multivariable models.
- For the variable "Do you now smoke cigarettes?", data from variables SMO020 and SMO040 were combined. Participants who reported smoking fewer than 100 cigarettes in their lifetime or responded "not at all" were coded as non-smokers.
- Dental visit: Defined as a visit to a dentist, dental hygienist, or dental clinic (NHANES protocol).
- Diabetes status: Defined as a self-reported medical history where a health professional had informed the participant of having diabetes, which includes prediabetes, impaired fasting glucose, or impaired glucose tolerance.
- For the multiple imputation procedure, biological plausibility filters were applied to the glycated haemoglobin variable (NHANES variable: LBXGH). Within the 2011–2012 training

cohort, valid HbA1c values were constrained to a lower limit of 3.60% and an upper limit of 17.80%. Equivalently, within the 2013–2014 validation cohort, the HbA1c distribution was restricted to a lower limit of 3.70% and an upper limit of 17.50%.

Supplementary Table S5. Characteristics and missing data patterns of study variables and auxiliary variables (NHANES 2011–2012; adults aged 30–50 years, dentate). Sample size in the training data: n = 1732.

Variable name	Role in analysis	Data type	Total *n*	Re-fused (7/77)	Don`t Know (9/99)	Not as-sessa-ble (99)	Blank/ Missing (.)	Valid *n* (%)
Gender	IV	Cat.	1732	0	0	0	0	100.0%
Race/Hispanic origin	A	Cat.	1732	0	0	0	0	100.0%
Education	A	Cat.	1732	0	0	0	0	100.0%
Self-rated oral health	A	Cat.	1732	0	1	0	0	99.9%
Last dentist visit	A	Cat.	1732	0	3	0	0	99.8%
Reason for dental visit	A	Cat.	1732	0	3	0	24	98.4%
Smoking status	A	Cat.	1732	0	1	0	0	99.9%
Diabetes status	A	Cat.	1732	0	2	0	0	99.9%
OHAROCGP	DV	Cat.	1732	0	0	0	0	100.0%
OHAROCDT	A	Cat.	1732	0	0	0	0	100.0%
OHAROCOH	A	Cat.	1732	0	0	0	0	100.0%
Age (years)	IV	Cont.	1732	0	0	0	0	100.0%
Glycohemoglobin (%)	A	Cont.	1732	0	0	0	73	95.8%
PD T02 distal/buccal	A	Cont.	1732	0	0	18	65	95.2%
PD T02 mesial/buccal	A	Cont.	1732	0	0	16	65	95.3%
PD T02 distal/lingual	A	Cont.	1732	0	0	8	66	95.7%
PD T02 mesial/lingual	IV	Cont.	1732	0	0	8	66	95.7%
PD T03 distal/buccal	IV	Cont.	1732	0	0	16	72	94.9%
PD T03 mesial/buccal	A	Cont.	1732	0	0	16	72	94.9%
PD T03 distal/lingual	A	Cont.	1732	0	0	10	73	95.2%
PD T03 mesial/lingual	A	Cont.	1732	0	0	6	73	95.4%
PD T14 distal/buccal	IV	Cont.	1732	0	0	34	71	93.9%
PD T14 mesial/buccal	A	Cont.	1732	0	0	22	71	94.6%
PD T14 distal/lingual	A	Cont.	1732	0	0	12	72	95.2%
PD T14 mesial/lingual	A	Cont.	1732	0	0	11	72	95.2%
PD T15 distal/buccal	A	Cont.	1732	0	0	43	71	93.4%
PD T15 mesial/buccal	A	Cont.	1732	0	0	34	71	93.9%
PD T15 distal/lingual	A	Cont.	1732	0	0	19	72	94.7%
PD T15 mesial/lingual	IV	Cont.	1732	0	0	13	72	95.1%
PD T18 distal/buccal	A	Cont.	1732	0	0	18	70	94.9%
PD T18 mesial/buccal	A	Cont.	1732	0	0	13	70	95.2%
PD T18 distal/lingual	A	Cont.	1732	0	0	23	70	94.6%
PD T18 mesial/lingual	IV	Cont.	1732	0	0	18	70	94.9%
PD T19 distal/buccal	A	Cont.	1732	0	0	14	59	95.8%
PD T19 mesial/buccal	IV	Cont.	1732	0	0	13	59	95.8%
PD T19 distal/lingual	A	Cont.	1732	0	0	21	59	95.4%
PD T19 mesial/lingual	A	Cont.	1732	0	0	17	59	95.6%
PD T30 distal/buccal	A	Cont.	1732	0	0	16	63	95.4%
PD T30 mesial/buccal	A	Cont.	1732	0	0	9	63	95.8%
PD T30 distal/lingual	IV	Cont.	1732	0	0	26	63	94.9%
PD T30 mesial/lingual	A	Cont.	1732	0	0	22	63	95.1%

PD T31 distal/buccal	A	Cont.	1732	0	0	15	69	95.2%
PD T31 mesial/buccal	A	Cont.	1732	0	0	12	69	95.3%
PD T31 distal/lingual	IV	Cont.	1732	0	0	31	69	94.2%
PD T31 mesial/lingual	A	Cont.	1732	0	0	26	69	94.5%

Note: *n* = 1732 (analytic training cohort). Values for 'Refused', 'Don't know', and 'Not assessable' reflect raw NHANES response codes, which were treated as missing in the analytic models. Auxiliary variables (e.g., education, smoking status) were included in the multiple imputation model (Supplementary Table S4) to satisfy the Missing at Random (MAR) assumption.

Abbreviations: A: auxiliary variable (for multiple imputation); DV: dependent variable; IV: independent variable. For further details, please refer to Supplementary Table S4.

Supplementary Table S6. Operationalisation and coding of dependent and independent variables in multivariable models.

Variable Name	Reference (X=0) vs. Model Indicators (X=1)	Coding Structure
**Binary teeth metric**		
(T02, T03, T14, T15, T18, T19, T30, T31)	Absent (X=0); Present (X=1)	0=Absent; 1=Present
**Periodontal site metric**		
(C02A, S03D, S14D, C15A, C18A, S19S, C30P, C31P)	Continuous site-specific pocket depth metric	-1 = Missing/Absent tooth locus; 0 to 5 = Continuous measurement in millimetres (mm)
**Gum disease/problem**	No gum disease/problem (X=0); A gum disease/problem (X=1)	0= No gum disease/problem; 1= A gum disease/problem
**Gender**	Female (X=0); Male (X=1)	0=Female; 1=Male
**Age** (years)	Continuous age metric	Modelled linearly per 1-year increment (Range: 30–50)

Note: Gum disease/problem served as the dependent variable (outcome) in all multivariable models presented in this research, including sensitivity analyses. All categorical independent variables were dichotomised; X = 1 indicators were compared against the designated reference category (X = 0) to derive adjusted odds ratios (aORs). Here, "dichotomised" refers specifically to the binary recoding shown in this Supplementary Table S6, not to the original categorical structures in the raw NHANES variables. Continuous variables were modelled linearly as specified in the coding matrix.

Supplementary Note S7: Full prediction model specification and access

The complete prediction model is fully specified across the main manuscript and the Supplementary Information material. Predictor definitions, coding rules, regression coefficients, and the model intercept are presented in Tables 1 and 2 of the main manuscript and in Supplementary Table S6. Together, these sources provide all the information required for the independent implementation of the model in any statistical software or programming environment, including SPSS, R, Python, Stata, and SAS.

Because the model was developed using R and no proprietary model object or scoring file was generated, no separate model object or syntax file is required for clinical implementation. Predictions can be computed directly by applying the regression equation using the coefficients and intercept reported in the manuscript.

The model specification is freely available for academic and non-commercial use. Users may implement the model without restriction, provided that the original publication is cited. No proprietary components or licensed algorithms beyond standard R procedures were used in model development. This Supplementary Note therefore confirms that the full model specification is openly available and reproducible based on the information provided in the main article.

Supplementary Note S8: Results of model updating and updated model performance

Model updating. This prediction model represents a newly developed model, and no model updating procedures (such as recalibration, coefficient revision, or re-estimation using external data) were performed. All reported results therefore reflect the original model as developed using the NHANES dataset.

Updated model specification. Because no updating was conducted, the final model specification corresponds directly to the development model. The full set of predictor coefficients, intercept, and the coding conventions used for dichotomised categorical predictors are provided in Table 2 in the main manuscript and in Supplementary Table S6.

Model performance. All performance metrics reported in the main manuscript represent the performance of the original, unupdated model. These include discrimination (e.g., C-statistic [AUC]), calibration (e.g., calibration plots), and overall performance and clinical utility (e.g., sensitivity, specificity, and Decision Curve Analysis [DCA]). No post-update performance metrics are applicable, as the model's original parameters were maintained throughout the evaluation process.

Interpretation. The model's performance has been rigorously evaluated through external validation. The model demonstrated good discriminative ability and satisfactory calibration on the external validation cohort. However, because transportability can vary across different healthcare settings, users should interpret individual risk estimates with caution when applying the model to populations that differ from the validation cohort. Future geographical or temporal validations may be beneficial to ensure the model remains calibrated as clinical practices or population characteristics evolve.

Supplementary Note S9: Cut-off selection and performance

Youden-optimal threshold and stability. The theoretically optimal threshold was derived by maximising Youden's Index (J) within the full training cohort (*n* = 1732). Across the complex survey and multiply imputed samples, the pooled mathematical optimum was 0.400, yielding a pooled Youden's index of 0.6490 (95 % CI: 0.5998–0.6982). At this threshold of 0.400, the pooled screening sensitivity within the external validation set (*n* = 1855) was 76.58% (95% CI: 67.32% – 83.87%) and the pooled specificity was 80.53% (95% CI: 76.13% – 84.31%). Pooled sensitivity and specificity estimates were calculated using Rubin's Rules to fully account for the within-imputation and between-imputation variances introduced by the multiple imputation process.

Rationale for the 0.367 threshold. While a cut-off of 0.400 represented the mathematical optimum, the final classification threshold for the screening model was a priori anchored at the baseline training prevalence of 0.367. Using the external validation dataset, this pre-specified cut-off of 0.367 yielded a pooled sensitivity of 78.39% (95% CI: 69.48% – 85.29%) and a specificity of 79.10% (95% CI: 74.55% – 83.04%). This decision was driven by critical clinical considerations: prioritising clinical utility, maximising screening sensitivity, and enhancing early case detection are paramount to ensure that individuals in need of care are not missed. This pre-specified 0.367 threshold was strictly maintained throughout model development and was subsequently evaluated using the independent external validation cohort.

Summary of comparative performance. Sensitivity analyses confirmed the stability of model discrimination. The a priori established 0.367 threshold was maintained as the primary cut-off, as it provided an optimal balance between net clinical benefit and the clinical mandate for high screening sensitivity. For exploratory comparison, increasing the cut-off to a standard threshold of 0.50 resulted in a pooled sensitivity of 70.59% (95% CI: 60.84% – 78.78%) and a pooled specificity of 84.52% (95% CI: 80.96% – 87.53%). These sensitivity analyses illustrate the explicit trade-offs associated with higher thresholds; all substantive performance conclusions were drawn from the external validation cohort, which served as the primary evaluation of model performance.

Supplementary Table S7. Expanded characteristics of the dentate cohort aged 30–50 years, NHANES 2011–2012 (*n* = 1732)

**Characteristic**	**Analytic Sample (*n* = 1732)**	**Gum disease/ problem (36.7%)**	**No gum disease/ problem (63.3%)**
**Categorical Variables**	**Pooled (%)**	**Pooled (%)**	**Pooled (%)**
Gender			
Female	50.1%	37.3%	57.5%
Male	49.9%	62.7%	42.5%
Race/Hispanic origin			
Non-Hispanic white	62.2%	49.7%	69.5%
Mexican American	10.7%	17.1%	7.0%
Non-Hispanic black	11.4%	15.7%	8.9%
Other Hispanic	7.2%	9.0%	6.2%
Other Race—Incl. Multi-Racial	8.5%	8.5%	8.4%
Education			
College graduate or above	36.1%	15.8%	47.8%
Some college or AA degree	28.2%	28.6%	27.9%
High school/GED	19.1%	26.7%	14.7%
9–11th (Incl. 12gr. no diploma)	11.5%	19.5%	6.9%
Less than 9th grade	5.1%	9.5%	2.6%
Do you now smoke cigarettes			
Non-smoker	77.7%	64.9%	85.1%
Smoker (Some days)	3.8%	5.6%	2.8%
Smoker (Every day)	18.5%	29.6%	12.1%
Doctor told you have diabetes			
No	93.6%	90.0%	95.6%
Borderline	1.8%	2.7%	1.3%
Yes	4.6%	7.3%	3.0%
**Binary Teeth Variables**	**Pooled (%)**	**Pooled (%)**	**Pooled (%)**
T02 present	87.2%	76.6%	93.3%
T03 present	87.2%	79.8%	91.5%
T14 present	86.1%	76.2%	91.9%
T15 present	86.6%	81.2%	89.7%
T18 present	85.4%	75.0%	91.4%
T19 present	79.7%	67.9%	86.6%
T30 present	81.1%	69.5%	87.7%
T31 present	86.0%	74.3%	92.7%
**Continuous Variables**	**Pooled Mean (SD)**	**Pooled Mean (SD)**	**Pooled Mean (SD)**
Age (yrs)	40.2 (6.2)	41.0 (5.9)	39.6 (6.3)
C02A	2.2 (0.9)	2.8 (1.0)	1.9 (0.7)
S03D	1.6 (0.9)	2.1 (1.0)	1.4 (0.6)
S14D	1.8 (1.0)	2.5 (1.1)	1.5 (0.7)
C15A	1.9 (0.9)	2.5 (1.0)	1.6 (0.6)
C18A	2.3 (0.9)	2.8 (1.0)	2.0 (0.7)
S19S	1.7 (0.8)	2.2 (0.9)	1.4 (0.7)

C30P	2.4 (1.1)	3.0 (1.2)	2.1 (0.8)
C31P	2.2 (1)	2.9 (1.1)	1.9 (0.8)
Number of teeth present (1-28)	24.8 (1.9)	24.0 (2.3)	25.2 (1.5)

Note: Values represent descriptive statistics (means with standard deviations [SD] or weighted percentages) pooled across 20 multiple-imputation cycles within the analytic cohort (n = 1732). To account for the complex, multistage probability sampling design of NHANES, analyses were performed using specialised survey design weights (wtmec2yr). The sample is restricted to participants aged 30–50 years. For the continuous dental variables (C02A–C31P), missing value codes (–1) were excluded prior to analysis. Further details are provided in Table 2 of the main article. Education categories: AA (associate/vocational), GED (high-school equivalency), 9–11th (incomplete high school), <9th (incomplete middle school). Doctor told you have diabetes: Includes prediabetes, impaired fasting glucose, or impaired glucose tolerance as informed by a health professional. Number of teeth present represents a continuous scale ranging from 1 to 28. Number of teeth present was calculated as a sum of individual tooth status variables (OHX02TC–OHX15TC and OHX18TC–OHX31TC). Teeth coding: '1' (present) if the original NHANES code was '2' (permanent tooth present) and '0' otherwise. Third molars (teeth 1, 16,17, and 32) were excluded.

Supplementary Table S8. Feature-level contributions to model discriminative ability (AUC/C-statistics) in the internal (NHANES 2011–2012) and external (NHANES 2013–2014) samples

Rank	Predictor Variable	Internal ΔAUC (AUC_{tot} = 0.894)	External ΔAUC (AUC_{tot} = 0.872)	Change in Rank
1	Tooth no. 31 Probing Depth and Presence	-0.0089	-0.0053	-2
2	Tooth no. 14 Probing Depth and Presence	-0.0081	-0.0032	-4
3	Tooth no. 19 Probing Depth and Presence	-0.0072	-0.0031	-4
4	Tooth no. 15 Probing Depth and Presence	-0.0064	-0.0066	+2
5	Tooth no. 18 Probing Depth and Presence	-0.0046	-0.0052	+1
6	Tooth no. 2 Probing Depth and Presence	-0.0036	-0.0005	-4
7	Tooth no. 30 Probing Depth and Presence	-0.0031	-0.0029	-1
8	Age (30-50 years)	-0.0026	-0.0038	+3
9	Gender	-0.0022	-0.0006	0
10	Tooth no. 3 Probing Depth and Presence	-0.0012	-0.0071	+9

Note: ΔAUC represents the unique contribution of each predictor feature to the total model discriminative ability, calculated by the incremental change in the AUC value. This incremental change was obtained by systematically omitting each predictor framework—including individual variables like age and gender, or harmonised dental features combining tooth presence and probing depth simultaneously—from the full model and computing the resulting reduction in AUC (ΔAUC). Change in Rank reflects the shift in the relative importance of predictors—determined by their unique ΔAUC contribution—when comparing the internal model (baseline) to the external validation cohort. A positive value (+) indicates that a predictor climbed in relative importance in the external model, a negative value (–) indicates a decrease in relative rank, and zero (0) denotes a stable rank across both models (Internal data: n = 1732; external data: n = 1855).

Supplementary Table S9. Association of predictors with the outcome in univariable and bivariable models (Training dataset, *n* = 1732; imputed NHANES 2011–2012)

Predictor Variable	Model type	OR (95% CI)	p	R2
**Demographics & Behaviours**				
Gender (female = 0, male = 1)	Univariable (Crude)	2.269 (1.769–2.911)	<0.001	0.051
Age (Continuous)	Univariable (Crude)	1.038 (1.012–1.064)	0.004	0.016
**Tooth-Specific Predictors**				
**Tooth 02**				
C02A	Bivariable (Pairwise)	3.167 (2.090–4.798)	<0.001	0.274
T02 (absent = 0, present = 1)	Bivariable (Pairwise)	0.006 (0.001–0.027)	<0.001	
**Tooth 03**				
S03D	Bivariable (Pairwise)	3.098 (2.361–4.065)	<0.001	0.232
T03 (absent = 0, present = 1)	Bivariable (Pairwise)	0.017 (0.008–0.036)	<0.001	
**Tooth 14**				
S14D	Bivariable (Pairwise)	3.022 (2.306–3.961)	<0.001	0.274
T14 (absent = 0, present = 1)	Bivariable (Pairwise)	0.010 (0.004–0.027)	<0.001	
**Tooth 15**				
C15A	Bivariable (Pairwise)	4.165 (2.922–5.937)	<0.001	0.284
T15 (absent = 0, present = 1)	Bivariable (Pairwise)	0.006 (0.002–0.023)	<0.001	
**Tooth 18**				
C18A	Bivariable (Pairwise)	3.419 (2.661–4.393)	<0.001	0.263
T18 (absent = 0, present = 1)	Bivariable (Pairwise)	0.004 (0.002–0.012)	<0.001	
**Tooth 19**				
S19S	Bivariable (Pairwise)	3.889 (2.850–5.307)	<0.001	0.268
T19 (absent = 0, present = 1)	Bivariable (Pairwise)	0.007 (0.003–0.018)	<0.001	
**Tooth 30**				
C30P	Bivariable (Pairwise)	2.386 (1.790–3.183)	<0.001	0.218
T30 (absent = 0, present = 1)	Bivariable (Pairwise)	0.015 (0.005–0.045)	<0.001	
**Tooth 31**				
C31P	Bivariable (Pairwise)	2.809 (2.298–3.432)	<0.001	0.280
T31 (absent = 0, present = 1)	Bivariable (Pairwise)	0.008 (0.003–0.019)	<0.001	

Note**:** *n* = 1732 (analytic training cohort). Predictor variables were evaluated using univariable (crude) logistic regression for gender and age, and bivariable (pairwise) logistic regression for tooth-specific pairings. Estimates were pooled across 20 multiple-imputation datasets using Rubin's rules, incorporating complex samples survey weights. The reference category for binary tooth presence indicators is Absent (0) and for gender is Female (0). For each univariable and bivariable model, the reported model fit statistic represents the pooled Design-Based Nagelkerke Pseudo-R^2 calculated using Rubin's rules for scalar parameters with design-adjusted t-distributions across the 20 imputation cycles based on model deviance relative to the null design. Age and periodontal measurements were

modelled as continuous variables. Detailed predictor definitions follow the structural criteria outlined in Table 1.

Clinical and mathematical interpretation of continuous scores: Within the bivariable pairwise framework, the continuous tissue loss indicators (i.e., C02A) and binary retention status (i.e., T02) operate as an interconnected clinical balance. For example, for Tooth 02, each 1 mm increase in tissue destruction increases the odds of a generalised periodontitis classification 3.167-fold. Crucially, when evaluating a tooth retained in the maximum diseased state (score = 5 mm) against the absolute tooth extraction reference group (Absent = 0, where C02A = -1), the 6-step incremental shift across the continuous scale (moving from -1 mm up to 5 mm) completely overrides the protective baseline coefficient (OR = 0.006), multiplying the overall screening risk by a factor of 6.05 ($0.006 \times 3.167^6 = 6.054$). This mathematically demonstrates that active, high-severity tissue destruction at a single locus conveys a substantially higher screening risk for “gum disease/problem” than a historical extraction event at the same site.

Supplementary Table S10. Sensitivity Analysis: Comparison of Complete-Case Analysis vs. Primary Imputed Model (NHANES 2011–2012)

Predictor Variable	Complete-Case (CCA) (*n* = 1546) OR (95% CI)	Primary Model (MI) (*n* = 1732) OR (95% CI)	Directional Consistency
C02A	1.766 (1.355–2.302)***	1.426 (1.017–1.999)*	Consistent
S03D	1.264 (0.973–1.643)	1.336 (0.972–1.837)	Consistent
S14D	1.657 (1.256–2.185)***	1.566 (1.132–2.165)**	Consistent
C15A	1.734 (1.192–2.523)**	2.056 (1.372–3.087)***	Consistent
C18A	1.982 (1.533–2.563)***	1.738 (1.278–2.376)***	Consistent
S19S	1.853 (1.370–2.505)***	1.821 (1.317–2.517)***	Consistent
C30P	1.522 (1.081–2.141)*	1.326 (0.895–1.966)	Consistent
C31P	1.811 (1.512–2.170)***	1.739 (1.346–2.248)***	Consistent
T02 (absent = 0, present = 1)	0.064 (0.031–0.134)***	0.152 (0.048–0.480)**	Consistent
T03 (absent = 0, present = 1)	0.702 (0.318–1.550)	0.514 (0.207–1.273)	Consistent
T14 (absent = 0, present = 1)	0.095 (0.036–0.252)***	0.114 (0.038–0.343)***	Consistent
T15 (absent = 0, present = 1)	0.323 (0.113–0.925)*	0.155 (0.040–0.607)**	Consistent
T18 (absent = 0, present = 1)	0.099 (0.030–0.323)***	0.118 (0.035–0.396)***	Consistent
T19 (absent = 0, present = 1)	0.065 (0.024–0.175)***	0.093 (0.033–0.269)***	Consistent
T30 (absent = 0, present = 1)	0.242 (0.067–0.882)*	0.364 (0.094–1.475)	Consistent
T31 (absent = 0, present = 1)	0.054 (0.022–0.135)***	0.093 (0.032–0.270)***	Consistent
Gender (female = 0, male = 1)	1.944 (1.310–2.885)***	2.098 (1.495–2.945)***	Consistent
Age (years)	1.033 (1.004–1.062)*	1.034 (1.006–1.062)*	Consistent

Note: Complex samples survey weights were applied independently within both CCA and MI frameworks to account for the respective data structures. * $p < 0.05$, ** $p < 0.01$, *** $p < 0.001$. CCA = complete-case analysis; MI = Multiple Imputation.

Supplementary Table S11. Multivariable logistic regression analysis of variables for predicting the outcome (External dataset, *n* = 1855, NHANES cycle 2013–2014)

Predictor Variable	B	S.E.	aOR (95% CI)	p
C02A	0.027	0.239	1.028 (0.643–1.641)	0.910
S03D	0.489	0.277	1.630 (0.948–2.803)	0.087
S14D	0.343	0.152	1.409 (1.046–1.898)	0.030
C15A	0.616	0.212	1.851 (1.221–2.806)	0.006
C18A	0.504	0.207	1.656 (1.104–2.484)	0.018
S19S	0.409	0.263	1.505 (0.899–2.520)	0.132
C30P	0.373	0.157	1.452 (1.067–1.975)	0.021
C31P	0.171	0.149	1.186 (0.885–1.588)	0.259
T02 (absent = 0, present = 1)	-0.268	0.964	0.765 (0.116–5.065)	0.782
T03 (absent = 0, present = 1)	-2.049	0.83	0.129 (0.025–0.656)	0.017
T14 (absent = 0, present = 1)	-1.260	0.501	0.284 (0.106–0.757)	0.014
T15 (absent = 0, present = 1)	-2.277	0.722	0.103 (0.025–0.423)	0.002
T18 (absent = 0, present = 1)	-1.833	0.699	0.160 (0.041–0.629)	0.010
T19 (absent = 0, present = 1)	-1.509	0.742	0.221 (0.052–0.946)	0.051
T30 (absent = 0, present = 1)	-1.598	0.523	0.202 (0.073–0.563)	0.003
T31 (absent = 0, present = 1)	-1.224	0.44	0.294 (0.124–0.697)	0.007
Gender (female = 0, male = 1)	0.314	0.119	1.368 (1.084–1.727)	0.008
Age (years)	0.048	0.015	1.049 (1.019–1.081)	0.002
Constant	2.448	0.803	–	0.002

Note: *n* = 1855 (external validation cohort). Estimates were pooled across 20 multiple imputation datasets using Rubin's rules. To account for the complex NHANES survey design and maintain correct variance estimation, all analyses were performed using the survey and mitools packages in R. Age and PD predictors were modelled as continuous variables; categorical coding follows Table 1 in the Main article. Statistical estimates (B, SE, aOR, 95% CI, and p-value) are reported exclusively for the multivariable framework to prioritise adjusted risk estimates. The reference category for tooth presence indicators is Absent (0) and for gender is female (0). To account for missing teeth (coded as -1 in PD data), models for continuous PD were adjusted for tooth presence, and vice versa. Model Fit: Pooled External Design-Based Nagelkerke Pseudo-R^2 = 0.501, calculated using Rubin's rules for scalar parameters with design-adjusted t-distributions across the 20 imputation cycles based on model deviance relative to the null design.

Supplementary Note S10: Clinical and decision-analytic calibration of the screening threshold

Evaluation of the Selected Clinical Decision Threshold (*Pt* = 0.33). This section details the theoretical and practical justifications for implementing a probability cut-off of 0.33 (*Pt* = 0.33) within the decision curve analysis framework [29]. Grounded in the decision-analytic principles established by Vickers, selecting a specific *Pt* serves as a formal mathematical mechanism to balance the clinical costs of unnecessary secondary assessments (false positives) against the diagnostic hazards of leaving active pathology undetected (false negatives). Traditional binary classifiers frequently default to a standard 0.50 threshold. However, in population-level screening workflows, such an elevated boundary is often excessively restrictive, aggressively prioritising specificity at the cost of missing a substantial proportion of individuals with true disease.

When clinical priorities shift towards maximising early case detection rather than minimising minor over-referrals, lowering the decision boundary below the conventional 0.50 level is highly advantageous. In this investigation, establishing the threshold at 0.33 transitions the screening strategy from a rigid statistical default to one governed by clinical utility. This specific value reflects a harm-to-benefit ratio where identifying a true case of disease is valued twice as highly as avoiding a false alarm (a 1:2 clinical ratio). To evaluate the empirical impact of this strategy on independent data, a dedicated sensitivity analysis was executed utilising the multiply imputed NHANES 2013–2014 replication cycle, isolating the full external validation cohort (*n* = 1855). Applying the baseline regression weights and intercept to this independent cohort allowed for a practical comparison of how effectively the 0.33 and 0.50 cut-offs capture individual periodontal conditions. The findings demonstrated that implementing the 0.33 threshold flags a significantly larger segment of the population in need of clinical evaluation compared to the 0.50 standard. This outcome aligns with the statistical principle that lower decision boundaries inherently maximise screening sensitivity. While

the model maintains stable performance metrics at the traditional 0.50 level, the 0.33 alternative offers a more proactive clinical filter by capturing a larger pool of true cases.

By adjusting the decision rule to 0.33, the model achieved a notable increase in diagnostic sensitivity for periodontal conditions without causing an unmanageable surge in false alarms. All statistical estimates were computed using normalised survey weights, with the corresponding confidence intervals derived via the Wilson score method featuring continuity correction. The precise numerical breakdowns are detailed below.

Viewed as a practical triaging tool, the 0.33 threshold introduces an optimal screening workflow. From a resource perspective, a clinician only needs to conduct approximately two additional baseline evaluations to confirm one true case of periodontitis. Furthermore, these extra clinical evaluations possess therapeutic merit of their own; even for patients eventually found to be disease-free, the clinical encounter provides definitive rule-out validation and valuable peace of mind. Implementing such a low-intensity, high-sensitivity filter fits perfectly into a comprehensive oral healthcare pathway, encouraging long-term patient engagement and preventive awareness.

Performance breakdown for the periodontal outcome component. The diagnostic advantages of anchoring the decision criteria at 0.33 are clearly illustrated when analysing its performance specifically for identifying periodontal tissue destruction. Adopting this uniform threshold secures excellent sensitivity while remaining entirely practical for routine dental care environments.

Gum disease or problem screening metrics. Flagging an individual as high-risk for a periodontal condition triggers a standard care protocol, which routinely encompasses detailed pocket depth charting, scaling and root planing, strict longitudinal monitoring, and personalised oral hygiene coaching. Because these clinical procedures require a distinct commitment of clinical time and dental resources, the screening tool must carefully balance high patient recall with the practical necessity of avoiding massive systemic over-referrals.

At the 0.33 threshold, the model achieved a pooled sensitivity of 80.50% (95% CI: 72.08%–86.89%), representing a statistically driven increase over the 78.39% (95% CI: 69.48%–85.29%) recorded at

the pre-specified 0.367 level. This ensures that the vast majority of active periodontal lesions are identified before irreversible alveolar bone loss occurs, while successfully maintaining the overall volume of referrals within a logistically feasible range for standard dental networks.

Supplementary Note S11: Detailed analysis of parameter drifts and methodological sensitivity

Tooth-level cohort comparison and parameter drift. To examine how the regression coefficients behave between the two separate population periods, the model parameters were compared from the 2011–2012 development data directly with the newly fitted model on the 2013–2014 external validation dataset (Supplementary Table S11). Minor statistical differences between these waves reflect normal epidemiological variation rather than flaws in the model structure. For example, the risk effects for certain back teeth pocket depths weakened and lost statistical significance in the separate external validation model:

- Tooth 31 (C31P): aOR = 1.19, $p = 0.2526$
- Tooth 02 (C02A): aOR = 1.03, $p = 0.9092$

Binary indicators for tooth presence successfully maintained their highly significant protective log-odds profiles across nearly all positions. Healthy tooth retention safeguarded the structural integrity of the baseline offset configurations.

Multiple imputation vs. Complete-case discrepancies. When comparing the primary pooled multiple imputation model against the raw data complete-case analysis (Supplementary Table S10), the adjusted training intercept reflected a minor scale translation, shifting from a pooled multiple imputation value of 3.793 to a complete-case baseline of 4.495. Fluctuations in statistical significance thresholds remained strictly localised and non-disruptive:

- Tooth 03 (S03D): This maxillary continuous molar pocket depth exhibited a highly consistent risk trend, remaining just outside the formal significance threshold ($p > 0.05$) in both the complete-case subset (OR = 1.264, 95% CI: 0.973–1.643) and the pooled multiple imputation sample (OR = 1.336, 95% CI: 0.972–1.837).
- Tooth 31 (C31P): This continuous tissue loss indicator (pocket depth) demonstrated powerful cross-methodological stability, maintaining strong statistical significance ($p < 0.001$) in both

the raw complete-case data (OR = 1.811, 95% CI: 1.512–2.170) and the adjusted multiple imputation matrix (OR = 1.739, 95% CI: 1.346–2.248).

- Tooth 30 (C30P & T30): Minor localised variations appeared at these sites, where marginal significance in the complete-case analysis ($p < 0.05$) attenuated to non-significance ($p > 0.05$) under the primary multiple imputation model for both the continuous pocket depth (C30P) and the binary tooth presence indicator (T30), while safely maintaining full directional consistency.

Feature importance and rank shifts between separate data models. To compare feature performance, two independent regression models were trained separately on their respective datasets. In the model built on the internal development data (NHANES 2011–2012; total AUC = 0.894; note that this internal model performance should not be confused with the main manuscript's locked external validation AUC estimates), the top three predictors were defined by the combined probing depth and tooth presence framework at specific sites (Supplementary Table S8):

- Tooth 31: ΔAUC = -0.0089 (Rank 1)
- Tooth 14: ΔAUC = -0.0081 (Rank 2)
- Tooth 19: ΔAUC = -0.0072 (Rank 3)

Upon fitting a separate, wave-specific regression model within the independent NHANES 2013–2014 validation population (total AUC = 0.872; note that this represents the internal performance of a newly fitted model and must not be confused with the primary locked model's external validation AUC reported in the main text), these specific anatomical sites decreased in relative importance: Tooth 31 shifted from Rank 1 (in 2011–2012) to Rank 3 (in 2013–2014), while both Tooth 14 and Tooth 19 shifted from Rank 2 and Rank 3 to Rank 6 and Rank 7 respectively. Conversely, in the 2013–2014 data, Tooth 15 ΔAUC = -0.0066) and Tooth 18 ΔAUC = -0.0052) climbed to Rank 2 (+2 ranks) and Rank 4 (+1 rank) respectively.

The biggest difference between the two separate models appeared at Tooth 03. It jumped up by +9 ranks, climbing from the very bottom of the internal model (Rank 10, ΔAUC = -0.0012) to become the single strongest predictor in the validation data model (Rank 1, ΔAUC = -0.0071). Simultaneously, general background factors behaved differently in each model: the impact of Age became stronger in the validation model (ΔAUC = -0.0038, climbing +3 ranks), while Gender remained completely stable at Rank 9 despite its minor absolute contribution (ΔAUC = -0.0006 in the validation data). (See detail in the Supplementary Table S8.)

Intercept and baseline probability comparison. The multivariable logistic regression model yielded a statistically significant training constant of 3.793 (SE = 0.828, $p < 0.001$). Upon re-estimating the model architecture within the independent 2013–2014 cohort, this baseline intercept was estimated at a constant value of 2.448 (SE = 0.803, $p = 0.002$). This mathematical difference corresponds to a baseline odds reduction, driven directly by the lower background disease prevalence in the NHANES 2013–2014 external validation cohort (32.69%) compared to the NHANES 2011–2012 development framework (36.71%; Supplementary Materials: Figure S1), while maintaining robust statistical significance.
